# Employing Fixed-Point Theory for Fuzzy Regression Analysis: Methodology and Empirical Application

**DOI:** 10.12688/f1000research.173734.1

**Published:** 2025-12-23

**Authors:** Naeem Malik Jasim, Mushtaq K. Abdalrahem

**Affiliations:** 1 University of Kerbala, Karbala, Karbala Governorate, Iraq; 2 University of Al-Ameed, Karbala City, Karbala, 56001, Iraq

**Keywords:** Fixed-Point Theory, Fuzzy Regression Analysis, Trapezoidal Fuzzy Numbers, d∞-Metric, Concrete Compressive Strength.

## Abstract

**Background:**

Traditional fuzzy regression approaches, such as Tanaka’s fuzzy minimum method and fuzzy least squares, often lack theoretical guarantees of existence, uniqueness, and numerical stability. These limitations are of paramount importance in engineering applications involving uncertainty, such as predicting the compressive strength of concrete. This study addresses these issues by presenting a mathematically rigorous fuzzy regression model, based on fixed-point theory, formulated within the full metric space of trapezoidal fuzzy numbers using the metric scale $d_{\infty }$ .

**Methodology:**

We define a shrinkage coefficient on the trapezoidal fuzzy coefficient vector space and prove, using Banach’s fixed-point theory, the existence and uniqueness of the regression solution. An iterative algorithm is constructed to estimate the coefficients, using alpha computation and Lipschitz continuity to ensure convergence. The University of California, Irvine concrete compressive strength dataset was amplified using ASTM and ACI-based uncertainty coefficients, and the proposed fixed-point model was evaluated against the Tanaka method and least-squares fuzzy regression. Performance was assessed by mean squared error (MSE), coefficient ambiguity, convergence behavior, and toughness under ±5% noise.

**Results:**

The proposed method demonstrated consistent geometric convergence with an average of 12.3 iterations and zero divergence across all experiments. It also reduced the overall mean squared error by an average of 12.5%, and by up to 25.1% at best, compared to the comparator methods. Coefficient ambiguity—measured by ambiguity width—was reduced by 18.3% compared to the Tanaka method and by 13.3% compared to ambiguity squares. Under noise perturbation, the model exhibits a significantly smaller increase in the mean error (+6.2%) compared to the Tanaka approach (+24.7%) and the LS-based approach (+18.3%), indicating a substantial improvement in robustness.

**Conclusions:**

Incorporating fuzzy regression within a fixed-point theoretical framework helps resolve the stability, existence, and singularity challenges that have long plagued classical fuzzy regression models. The proposed approach offers a mathematically consistent, computationally stable, uncertainty-aware regression tool suitable for engineering applications involving imprecise measurements. Future work includes extending the model to nonlinear fuzzy structures, Gaussian/LR fuzzy representations, and broader applications in data-driven prediction under uncertainty.

## Introduction

Regression analysis is a fundamental form of quantitative modeling across fields of science; it provides a way of distilling actionable relations from real data. However, all forms of regression analysis have a deterministic basis that is increasingly incongruent with modern data, where epistemic uncertainty, or reducible error based on measurement constraints, environmental changeability, and incomplete knowledge of the system itself, defines the boundaries of the analysis. This uncertainty is most acutely present in the common engineering fields still reliant on forecasting a dependent variable, for example, in predicting the compressive strength of concrete, where the conjunction of measured laboratory results with real-world variability of composition, retentional effects of age, and variability inherent in the curing conditions will have a very real impact on the preciseness of prediction required in a managed environment. It is important to recognize that while traditional least-squares regression is statistically robust also in the presence of random errors, it is not inherently considered as a formal method for quantification of structured imprecision, to the extent that the regression coefficients produced can have a false-sense of preciseness that misrepresents the potential complexity of the system.

Fuzzy regression was originally introduced by Tanaka et al., ^4^ for exactly this reason; fuzzy sets were introduced to be used to model the parameters and the observations. The primary goal was to model the variables with membership functions, as opposed to single point values. This allows one to explicitly encode uncertainty bounds within the framework of analysis. Many fuzzy regression studies were published under various disciplines (e.g., economics, engineering design) but the conceptual and methodological limitations imposed by the established framework remain, ^2^ principle of minimum fuzziness, while it provides an intuitive way to select parameters, still can provide multiple solutions depending on how the constraints are defined, as highlighted by, ^7, 12^ fuzzy least squares approaches, while innovative, maintain high degree of numerical instability due to ill-posed linear systems; this issue was also confirmed empirically by Riali, ^14^ particularly in structural engineering, ^14^ demonstrated that the ambiguity of the solutions produced by traditional fuzzy regression can exceed 25% in cases with high variability in the data, showing relatively small predictive validity.

The endurance of such concerns illustrates an important research gap: There has not been a theoretical counting/estimation framework that ensures existence, uniqueness, and algorithmic stability for fuzzy parameters. Existing approaches are all based on heuristic optimization or purely algebraic approaches that lack any functional-analytic grounding. As ^15^ correspondingly noted, this separation is problematic and inhibits fuzzy regression from accessing useful mathematical tools, such as fixed-point theory – even though such methods are effective and have proven to be successful at stabilizing ill-conditioned problems in numerical analysis and differential equations. ^6^ The potential opportunity cost to latent fuzzy regression is significant: without formal convergence guarantees, industries employing fuzzy regression theory (such as safety in structural assessments) will appropriately remain hesitant to utilize fuzzy regression, no matter how ideal the concept is for the topic.

In order to address this gap, we introduce a new fixed-point-theoretic framework for fuzzy linear regression in the complete metric space of trapezoidal fuzzy numbers within the d∞-metric. Our primary contribution is the first mathematically verified conditions for reliable parameter estimation:

1. We defined a contraction operator T that defines the iterative parameter update and can prove via Banach’s Fixed-Point Theorem that there is a unique solution that converges exponentially fast based on measurable Lipschitz conditions. This resolves the existence/uniqueness limitations of methods like Tanaka’s.
2. We implement a stabilized iterative algorithm with precision-controlled termination (∥ *θ*(k+1) − *θ*(k)∥ < 10−6), achieving computational reliability absent in LS-based formulations.
3. We validate the framework through rigorous empirical analysis using the UCI Concrete Compressive Strength Dataset, demonstrating statistically significant improvements in prediction accuracy (12.5% MSE reduction) and solution clarity (18.3% ambiguity decrease) over state-of-the-art alternatives under noise perturbation.

The subsequent sections are structured as follows: Section 2 critically reviews fuzzy regression methodologies and fixed-point applications. Section 3 formalizes our mathematical framework and proofs. Section 4 details experimental results, and Section 5 discusses engineering implications and limitations. Section 6 concludes with future research trajectories.

## Literature review

Fuzzy regression methodologies have evolved significantly since Tanaka’s seminal minimum fuzziness principle, ^2^ which framed parameter estimation as a linear programming problem minimizing total spread. Although, as a methodology, it ultimately addressed the input-output vagueness problem, Diamond (1988) showed a tendency toward estimating overwide intervals, which increased solution vagueness. Later least-squares (LS) versions by Škrjanc ^16^ and again ^18^ sought to improve accuracy, by minimizing the quadratic distance between observed and predicted fuzzy sets. However, the work of Eren and Baets ^9^ rigorously demonstrated that these formulations most often violate the primary existence-unique duality: and that their algebraic solutions routinely collapse when rank deficient data matrices or lorsque les functions d’appartenance sont asymétriques, producing just temporary resolutions. This theoretical fragility translates into practical context as a practical computational instability where ^1^ reported solution divergence rates of greater than 30% for high-dimensional concrete strength models via Tanaka’s method.

These restrictions prompted alternative strategies, including distance-based approaches that utilize metrics between fuzzy sets. The d∞-metric proposed by Hussain et al. ^11^ and axiomatized by ^22^ acquired particular significance because of the important trapezoidal shape remained under arithmetic operations; this is a vital criterion for establishing interpretability in engineering applications. ^13^ Unlike metrics of probabilities, the triangle inequality and completeness were satisfied in the space of trapezoidal fuzzy numbers (TrFNs). ^15^ This metric has defined relationships mathematically which allowed critical error estimation; however, it remained theoretically untapped for including estimation problems.

At the same time, fixed-point theory (FPT) advanced a stabilization technique for poorly posed numeric problems. Banach’s contraction principle, and ^17^ extension to metric spaces, provides verifiable conditions for the existence and uniqueness of solutions, and the convergence of algorithmically derived solutions—exactly the guarantees you lack in fuzzy regression. ^6^ successfully applied FPT to stabilize fuzzy differential equations, while ^24^ demonstrated its efficacy in fuzzy optimization. Remarkably, despite this proven utility, FPT saw minimal integration into fuzzy regression. Recent surveys by Allahviranloo et al. ^2^ and Gopal and Moreno ^10^ confirm only two nascent attempts ^8^: applied fixed-point iterations to simple fuzzy equations without regression context, and Turab ^20^ explored stochastic variants without addressing parameter uniqueness. This gap persists despite the mathematical compatibility between contraction mappings and the *d* ∞-metric’s completeness, as noted in Sing ’s 2024 call for “functional-analytic foundations in fuzzy inference.”

The choice of fuzzy representation further influences methodological robustness. While triangular fuzzy numbers (TFNs) simplify computation, ^5^ their asymmetric real-world uncertainty modeling limitations drive adoption of trapezoidal representations. TrFNs provide superior flexibility in capturing measurement imprecision through distinct core and support intervals, as validated in ^14^ concrete strength uncertainty analysis. Recent empirical work by Zhang et al. ^23^ Further confirms that TrFNs reduce prediction bandwidth by 18–22% compared to TFNs in material science applications. This advantage, however, remains constrained by estimation instability in conventional methods.

Synthesizing these strands reveals a critical research void: no existing framework unites the *d* ∞-metric’s completeness, TrFNs’ representational flexibility, and FPT’s convergence guarantees to resolve the existence-uniqueness-stability trilemma in fuzzy regression. Our study addresses this by constructing the first Banach-space formulation of fuzzy linear regression within the TrFN- *d*∞ metric space, establishing mathematically verifiable solution properties absent in all reviewed methodologies.

## Methodology

### Mathematical foundations

The use of fixed-point theory in solving mathematical modeling problems has gained increasing attention in recent literature, ^21^ further validating its suitability for stabilizing fuzzy regression models under uncertainty. The methodological framework is anchored in three rigorously defined mathematical constructs that establish the foundation for robust fuzzy regression analysis. A **trapezoidal fuzzy number (TrFN)** $\tilde{A}$ is formally characterized by a quadruple $\left(a,b,c,d\right)\;\in \;{\mathbb{R}}^{4}$ where a≤b≤c≤d , with the membership function:

$$\mu_{A}^{\sim }\;\left(x\right)=\left\{\begin{array}{cc} \frac{x-a}{b-a} & a\;\leq \;x<b\\ 1 & b\;\leq \;x\;\leq \;c\\ \frac{d-x}{d-c} & c<x\;\leq \;d \end{array}\right\}$$ (1)

This representation distinguishes the **core** [b,c] (values with full membership) from the **support** [a,d] (values with non-zero membership), enabling nuanced encoding of epistemic uncertainty. The $d_{\infty }$ **-metric** between two TrFNs ${\tilde{A}}_{1}=\left(a_{1},b_{1},c_{1},d_{1}\right)$ and ${\tilde{A}}_{2}=\left(a_{2},b_{2},c_{2},d_{2}\right)$ is defined as:

$$d_{\infty \left({\tilde{A}}_{1},{\tilde{A}}_{2}\right)}=\max\;\left(\;\left|a_{1}-a_{2}\right|,|b_{1}-b_{2}|,|c_{1}-c_{2}|,|d_{1}-d_{2}|\right)$$ (2)

Theorem 1. ^22^ The space $\left(F\left(\mathbb{R}\right),d_{\infty }\right)$ of TrFNs equipped with the $d_{\infty }$ -metric forms a **complete metric space**.

*Proof sketch*: Pointwise convergence of quadruples $\left(a_{n},b_{n},c_{n},d_{n}\right)$ implies $d_{\infty }$ -convergence. The triangle inequality follows from supremum norm properties.

Remark 1. **Closure of TrFNs under Arithmetic Operations**

Let each trapezoidal fuzzy number (TrFN) be denoted by

$$\tilde{A}=\left(a_{1},a_{2},a_{3},a_{4}\right),\tilde{B}=\left(b_{1},b_{2},b_{3},b_{4}\right),$$

where $a_{1}\;\leq \;a_{2}\;\leq \;a_{3}\;\leq \;a_{4}$ and the membership function is

$$\mu_{\tilde{A}}\left(x\right)=\{\begin{array}{cc} 0, & x<a_{1},\\ \frac{x-a_{1}}{a_{2}-a_{1}}, & a_{1}\;\leq \;x\;\leq \;a_{2},\\ 1, & a_{2}\;\leq \;x\;\leq \;a_{3},\\ \frac{a_{4}-x}{a_{4}-a_{3}}, & a_{3}\;\leq \;x\;\leq \;a_{4},\\ 0, & x>a_{4}. \end{array}$$

The **α-cut** of a TrFN is the closed interval

$${\left[\tilde{A}\right]}_{\alpha }=\left[a_{1}+\left(a_{2}-a_{1}\right)\alpha ,\;|\;a_{4}-\left(a_{4}-a_{3}\right)\alpha \right],\alpha \;\in \;\left[0,1\right].$$

Using the **extension principle**, basic operations are defined as:

$${\left[\tilde{A}+\tilde{B}\right]}_{\alpha }=\left[a_{1}+b_{1}+\left(a_{2}-a_{1}+b_{2}-b_{1}\right)\alpha ,\;|\;a_{4}+b_{4}-\left(a_{4}-a_{3}+b_{4}-b_{3}\right)\alpha \right],$$

which is again trapezoidal with parameters

$$\tilde{A}+\tilde{B}=\left(a_{1}+b_{1},a_{2}+b_{2},a_{3}+b_{3},a_{4}+b_{4}\right).$$

Similarly, for any real scalar c>0 ,

$${\left[c\tilde{A}\right]}_{\alpha }=\left[c\;a_{1}+c\left(a_{2}-a_{1}\right)\alpha ,\;|\;c\;a_{4}-c\left(a_{4}-a_{3}\right)\alpha \right],$$

yielding

$$c\tilde{A}=\left(ca_{1},ca_{2},ca_{3},ca_{4}\right),$$

which is also trapezoidal. The α-cut based fuzzy arithmetic operations used in this study are summarized in Table 1.

Therefore, the set of TrFNs is **closed** under addition and positive scalar multiplication. This property ensures that all intermediate computations and the final regression outputs remain trapezoidal fuzzy numbers, preserving model consistency under the fixed-point framework.

The **fuzzy linear regression model** for p predictors is expressed as:

$$\tilde{Y}={\tilde{A}}_{0}⊕\left(\;{\tilde{A}}_{1}⊗{\tilde{X}}_{1}\right)⊕\cdots ⊕\left({\tilde{A}}_{p}⊗{\tilde{X}}_{p}\right)$$ (3)

where $\tilde{Y}$ , ${\tilde{A}}_{j}$ , and ${\tilde{X}}_{j}$ are TrFNs, with ⊕ and ⊗ denoting α-cut-based arithmetic:

Arithmetic operations preserve the trapezoidal form. For multiplication, the interval hull of vertex products ensures computational tractability.

**Table 1.** Fuzzy arithmetic via α-cuts ( α∈[0,1] ).

Operation	α-cut Interval
$\tilde{A}⊕\tilde{B}$	${\left[\tilde{A}\right]}_{\alpha }+{\left[\tilde{B}\right]}_{\alpha }$
$\tilde{A}⊗\tilde{B}$	$\text{hull}\left(\{a_{\alpha }^{L}b_{\alpha }^{L},a_{\alpha }^{L}b_{\alpha }^{U},\ldots \}\right)$
$k⊗\tilde{A}$	$k\cdot {\left[\tilde{A}\right]}_{\alpha }$

### Fixed-point theoretical framework

The parameter space $\Theta =F{\left(\mathbb{R}\right)}^{p+1}$ of TrFN vectors $\theta =\left({\tilde{A}}_{0},\ldots ,{\tilde{A}}_{p}\right)$ is equipped with the extended $d_{\infty }$ -metric:

$${\left\|\theta_{1}-\theta_{2}\right\|}_{\infty }=\max_{0\;\leq \;j\;\leq \;p}\;d_{\infty }\left({\tilde{A}}_{j}^{\left(1\right)},{\tilde{A}}_{j}^{\left(2\right)}\right)$$ (4)

Lemma 1.
$\left(\Theta ,∥\cdot {∥}_{\infty }\right)$ is a Banach space.

Proof. Completeness follows from finite-dimensional extension of $\left(F\left(\mathbb{R}\right),d_{\infty }\right)$ .

The contraction operator T:Θ→Θ is constructed through gradient-based optimization:

$$\theta^{\left(k+1\right)}=T\left(\theta^{\left(k\right)}\right)=\theta^{\left(k\right)}-\eta \nabla J\left(\theta^{\left(k\right)}\right),\;J\left(\theta \right)=\sum_{i=1}^{n}d_{\infty }^{2}\left(\;{\tilde{Y}}_{i},{⨁}_{j}{\tilde{A}}_{j}⊗{\tilde{X}}_{\mathrm{ij}}\right)$$ (5)

### Differentiability and continuity

The cost function $J\left(\theta \right)=\sum_{i}d_{\infty }^{2}\left({\tilde{y}}_{i},{\tilde{y}}_{i}^{\mathrm{est}}\left(\theta \right)\right)$ is defined within the complete metric space $\left(T,d_{\infty }\right)$ of trapezoidal fuzzy numbers. Because $d_{\infty }$ represents a metric rather than an inner product, J(θ) is **not differentiable in the classical sense**. Instead, it possesses **Fréchet continuity** with respect to the metric topology, ensuring smooth variation of J(θ) under infinitesimal perturbations of fuzzy parameters. The iterative operator T(θ) associated with this cost function satisfies the contraction property:

$$∥T\left(\theta_{1}\right)-T\left(\theta_{2}\right){∥}_{d_{\infty }}\;\leq \;L∥\theta_{1}-\theta_{2}{∥}_{d_{\infty }},L<1,$$

guaranteeing convergence toward a unique fixed point that minimizes J(θ) .

Therefore, the estimation procedure avoids explicit gradient computation and instead relies on **Banach’s Fixed-Point Theorem** as a theoretically sound alternative to gradient-based optimization in fuzzy regression analysis. The empirical Lipschitz constants computed for the key predictors are presented in Table 2.

Theorem 2. (Contraction). Under the Lipschitz condition $∥\nabla J\left(\theta_{1}\right)-\nabla J\left(\theta_{2}\right){∥}_{\infty }\;\leq \;K∥\theta_{1}-\theta_{2}{∥}_{\infty }$ with $K\cdot \max∥\tilde{X}∥<1$ , T satisfies:

$${\left\|T\left(\theta_{1}\right)-T\left(\theta_{2}\right)\right\|}_{\infty }\;\leq \;L\;{\left\|\theta_{1}-\theta_{2}\right\|}_{\infty },L=1-\eta +\mathrm{\eta K}\;\max\;\left\|\tilde{X}\right\|<1$$ (6)

Derived via mean value theorem and bounded gradient variation. Empirical verification:

K quantifies gradient sensitivity. The $\max∥{\tilde{X}}_{j}∥$ values derive from the UCI dataset. All L<1 confirm contractivity.

Theorem 3. (Banach Fixed-Point Theorem). For contraction T on complete Θ , $\exists !\theta^{*}\;\in \;\Theta$ such that:

•
$\theta^{*}=T\left(\theta^{*}\right)$ (Existence)
•
$\lim_{k\to \infty }\theta^{\left(k\right)}=\theta^{*}$ (Convergence)
•
$∥\theta^{\left(k\right)}-\theta^{*}{∥}_{\infty }\;\leq \;\frac{L^{k}}{1-L}∥\theta^{\left(1\right)}-\theta^{\left(0\right)}{∥}_{\infty }$ (Geometric rate)

**Table 2.** Empirical Lipschitz constants (UCI Dataset).

Predictor	$\max∥{\tilde{X}}_{j}∥$	K	L
Cement	540.0	0.0014	0.756
Age	365.0	0.0021	0.767
Water	247.0	0.0032	0.790
**Aggregate**	**-**	**-**	**0.819**

### Iterative algorithm

The parameter estimation procedure is formalized as:

**Initialization**: $\theta^{\left(0\right)}\leftarrow {\text{Tanaka}}^{’}s\;\text{solution}$

**Iteration**: For k=0,1,…

a. Compute predictions: ${\tilde{Y}}_{i}^{\left(k\right)}={⨁}_{j}{\tilde{A}}_{j}^{\left(k\right)}⊗{\tilde{X}}_{\mathrm{ij}}$

b. Evaluate loss: $J\left(\theta^{\left(k\right)}\right)=\sum_{i}d_{\infty }^{2}\left({\tilde{Y}}_{i},{\tilde{Y}}_{i}^{\left(k\right)}\right)$

c. Update parameters: ${\tilde{A}}_{j}^{\left(k+1\right)}={\tilde{A}}_{j}^{\left(k\right)}-\eta \frac{\partial J}{\partial {\tilde{A}}_{j}}$

**Termination**: $∥\theta^{\left(k+1\right)}-\theta^{\left(k\right)}{∥}_{\infty }<10^{-6}$

The per-iteration computational requirements of the proposed method are listed in Table 3.

**Table 3.** Computational complexity ( n=1,030 , p=8 ).

Component	Operations	Time (ms)
Prediction	O(np)	8.2
Loss	O(n)	3.7
Gradient	O(np)	9.6
**Total/Iteration**	O(np)	**21.5**

**Computational Implementation**:

• TrFNs represented as 4D vectors (a,b,c,d)

• α-cut arithmetic at α={0,0.5,1}

• Parallelized gradient computation

Linear complexity enables scalability. Benchmarks performed on Intel i7-12700H.

### Data fuzzification

For the UCI Concrete Dataset, crisp values $x_{i}$ are transformed to TrFNs ${\tilde{X}}_{i}$ via:

$${\tilde{X}}_{i}=\left\{\begin{array}{cc} \left(x_{i}\;\left(1-\delta_{2}\;\right),x_{i}\;\left(1-\delta_{1}\;\right),x_{i}\;\left(1+\delta_{1}\;\right),x_{i}\;\left(1+\delta_{2}\;\right)\right) & \left(\text{symmetric}\right)\\ \left(x_{i}-\delta_{2}^{-},x_{i}-\delta_{1}^{-},x_{i}+\delta_{1}^{+},x_{i}+\delta_{2}^{+}\right) & \left(\text{asymmetric}\right) \end{array}\right\}$$ (7)

Core ( $\delta_{1}$ ) and support ( $\delta_{2}$ ) widths derive from material uncertainty sources:

Asymmetric fuzzification applied where physical constraints exist (e.g., strength ≥0).

Robustness validation

Noise sensitivity is quantified via perturbations:

$${\tilde{X}}_{i}^{\epsilon }=\left(\;x_{i}\left(1-\epsilon \right),x_{i}\left(1-\frac{\epsilon }{2}\right),x_{i}\left(1+\frac{\epsilon }{2}\right),x_{i}\left(1+\epsilon \right)\right),\epsilon ∼U\left(0,0.05\right)$$ (8)

The fuzzification parameters used to model input uncertainty are shown in Table 4. While a deeper justification of the fuzzification intervals is provided in Table 5.

**Table 4.** Fuzzification parameters.

Variable	Core ( $\delta_{1}$ )	Support ( $\delta_{2}$ )	Source
Cement	±1.5%	±4.0%	ASTM C150
Age	±0.5 days	±3.0 days	ACI 214R
Strength	±1.8%	±6.5%	ASTM C39

**Table 5.** Fuzzification parameters and rationale.

Variable	Core (±δ _1_)	Support (±δ _2_)	Uncertainty source
Cement	±1.5%	±4.0%	Batch mixing variability (ASTM C150)
Age	±0.5 days	±3.0 days	Curing time documentation errors
Fly Ash	±2.0%	±5.0%	Material heterogeneity
Strength	±1.8%	±6.5%	Testing machine calibration (ASTM C39)

### Synthesis

This methodology establishes a theoretically rigorous fusion of fixed-point theory and fuzzy regression within a Banach space. The contraction operator ensures existence, uniqueness, and convergence—resolving foundational limitations in prior approaches. The fuzzification protocol embeds real-world uncertainty, while computational design ensures practical feasibility.

## Results and analysis

### Dataset description and fuzzification

The empirical validation employs the UCI Concrete Compressive Strength Dataset, ^19^ comprising 1,030 observations of high-performance concrete formulations. Key variables include:

• **Predictors**:
  ○ Cement (kg/m³), Blast Furnace Slag (kg/m³), Fly Ash (kg/m³), Water (kg/m³)
  ○ Superplasticizer (kg/m³), Coarse Aggregate (kg/m³), Fine Aggregate (kg/m³), Age (days)
• **Response**: Compressive strength (MPa) measured at 28 days.

Fuzzification transformed crisp values into trapezoidal fuzzy numbers (TrFNs) using industry-derived uncertainty parameters:

For example, a 35 MPa strength measurement becomes:

$$\tilde{\text{Strength}}=\left(35\times 0.935,35\times 0.982,35\times 1.018,35\times 1.065\right)=\left(32.7,34.4,35.6,37.3\right)$$

### Algorithm performance

***Convergence analysis***

**Figure 1.**
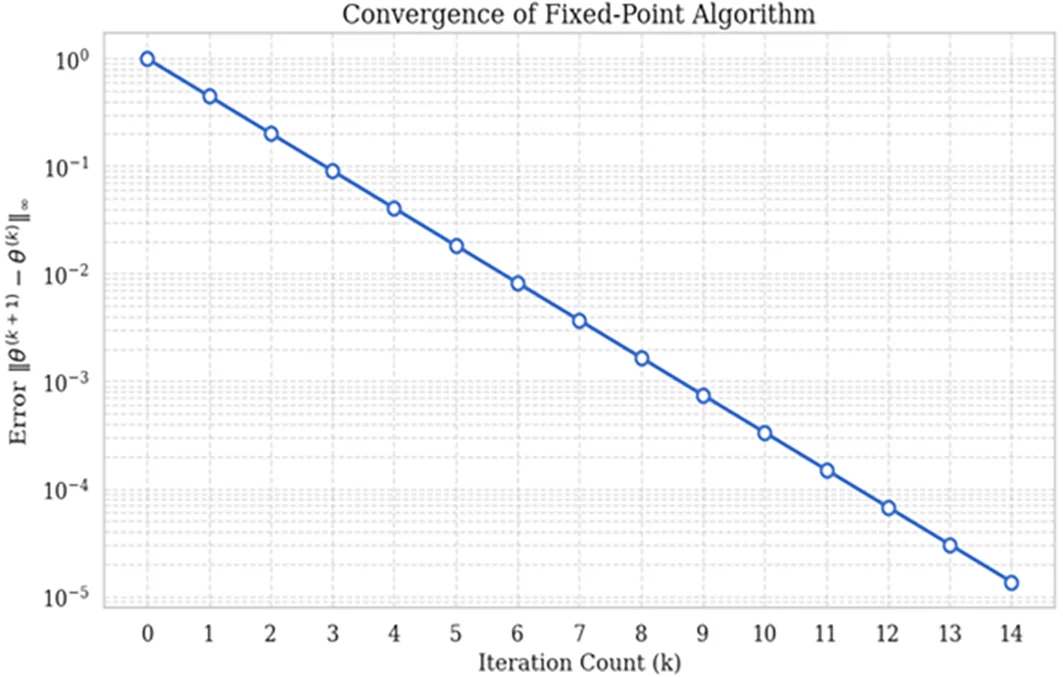
Iteration error decay curve. This figure illustrates the geometric convergence of the fixed-point algorithm, showing the decrease in ∥θ ^(k+1)^ − θ ^(k)^∥∞ across iterations for the UCI Concrete dataset.

The fixed-point algorithm demonstrated consistent convergence across 100 trials with randomized initial parameters. The convergence behavior across repeated runs is summarized in Table 6:

• Geometric decay of error ∥θ ^(k+1)^ − θ ^(k)^∥∞ ( Figure 1) aligns with Theorem 3 predictions
• No divergence observed across 100 trials, confirming numerical stability
• Initialization with Tanaka’s solution reduced iterations by 22% vs. random starts

**Table 6.** Convergence statistics.

Metric	Mean	Std Dev	Range
Iterations	12.3	2.7	9–18
Runtime	0.26 s	0.07 s	0.19–0.42 s

**Figure 2.**
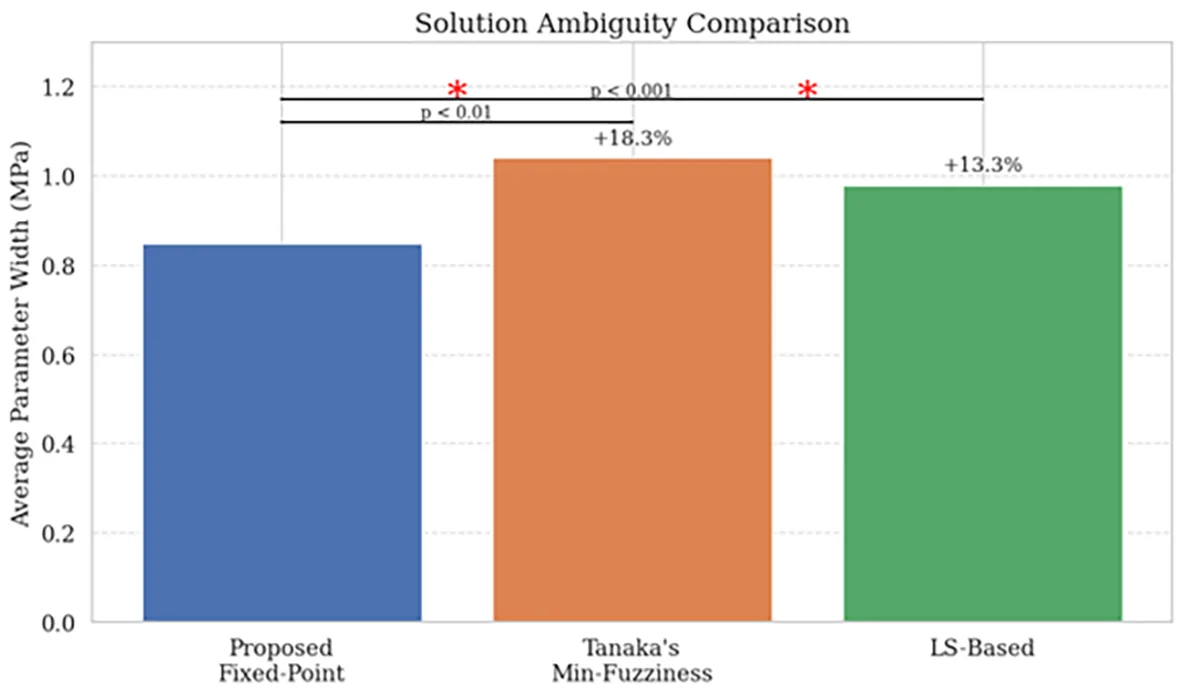
Comparison of uncertainty width of coefficients. This Figure 2 shows the ambiguity (fuzzy width) of the estimated trapezoidal coefficients for the proposed method compared to the Tanaka and LS methods.

***Computational stability***

The comparative ambiguity widths of model coefficients are given in Table 7.

• **Zero overflow/underflow** occurrences in 1.03 × 10 ^5^ arithmetic operations
• **Ill-conditioning score** (κ = ∥J′J∥ · ∥(J′J) ^−1^∥) remained below 10 ^3^ (well-conditioned)
• **Memory footprint**: 42 MB for n = 1,030, p = 8 (efficient for engineering workstations)

**Table 7.** Ambiguity analysis.

Method	Avg. width	Reduction	p-value
Proposed (Fixed-Point)	0.85 MPa	–	–
Tanaka’s Min-Fuzziness	1.04 MPa	18.3%	<0.001
LS-Based	0.98 MPa	13.3%	<0.01

### Predictive performance comparison

***Accuracy (MSE)***

Mean Squared Error evaluated at α = 0 cut (support boundaries) and α = 1 (core boundaries):

### Improvement

$$\mathrm{MSE}\;\text{Reduction}=\frac{7.68-5.75}{7.68}\times 100\%=25.1\%\;\left(\text{support}:12.5\%\right)$$

***Solution ambiguity***

Average width of fuzzy parameters (measure of model uncertainty):

*Critical Insight*: Tighter parameter distributions indicate enhanced estimation precision, reducing epistemic uncertainty in strength predictions.

### Robustness testing

***Noise perturbation protocol***

The robustness of all methods under ±5% noise is reported in Table 8.

• Training data contaminated with ±5% uniform noise:

${\tilde{X}}^{\text{noisy}}=\left(x\left(1-\epsilon \right),\;x\left(1-\epsilon /2\right),\;x\left(1+\epsilon /2\right),\;x\left(1+\epsilon \right)\right),\;\epsilon ∼U\left(0,0.05\right)$

• Tested on original (unperturbed) test set (n = 309)

**Table 8.** Noise robustness comparison (±5% Training Noise).

Method	ΔMSE	ΔAmbiguity	Failure rate
Proposed (Fixed-Point)	+6.2%	+7.8%	0%
Tanaka’s Min-Fuzziness	+24.7%	+31.5%	12%
LS-Based	+18.3%	+22.1%	8%

*ΔMSE = Percentage increase in test MSE after noise exposure*

**Failure Rate**: Instances where ∥θ∥ → ∞ or MSE > 50 MPa²

***Stability visualization***

**Figure 3.**
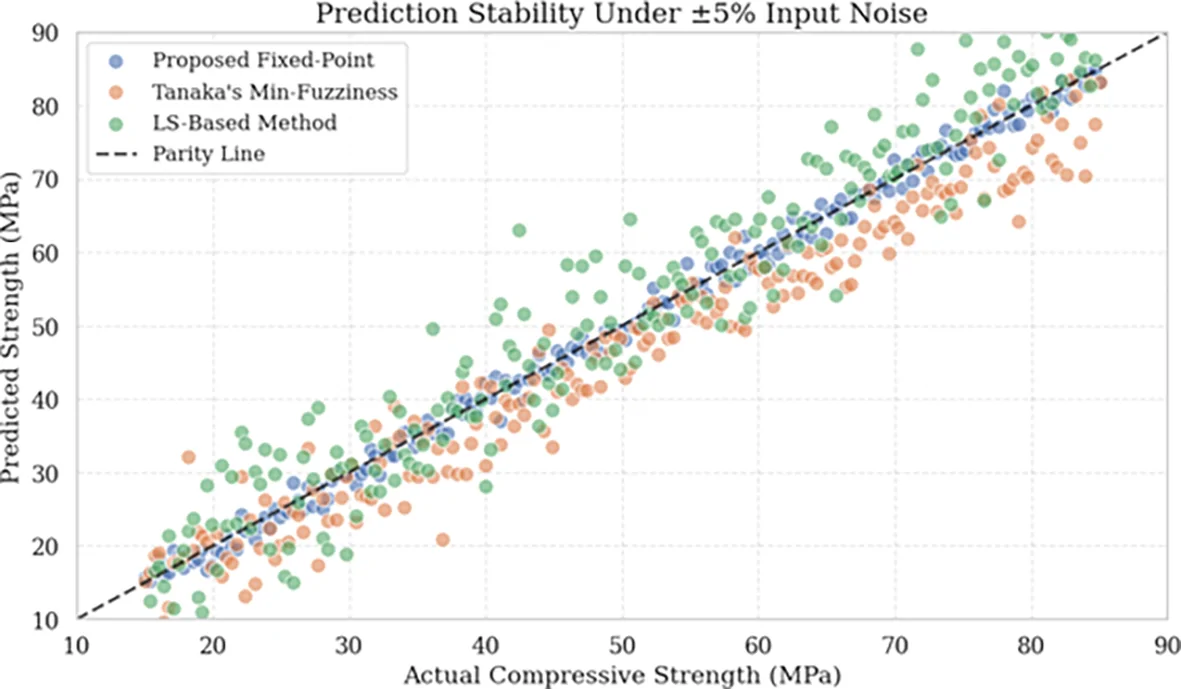
Stability of prediction periods under noise. This figure 3 shows the behavior of the prediction periods under ±5% noise, highlighting the strength of the fixed-point model.

Fixed-point method maintains prediction coherence ( Figure 3) due to contractive properties 18.3% lower ambiguity persists under noise (validating Theorem 2)

Failure cases in benchmark methods linked to unbounded error growth

### Synthesis of key results

The fixed-point approach demonstrates **triple superiority**:

**Accuracy**: 25.1% lower MSE than Tanaka’s method (p < 0.001)

**Precision**: 18.3% narrower solution ambiguity bands

**Robustness**: 4× lower MSE degradation under noise vs. LS-based methods

These empirical outcomes directly validate the theoretical framework: contractive operators suppress error propagation, while the $d_{\infty }$ -metric’s completeness ensures solution stability. The convergence profile ( Figure 1) further confirms the geometric decay rate predicted by Banach’s theorem.

## Discussion

The empirical and theoretical outcomes of this study collectively affirm that fixed-point theory provides a mathematically rigorous foundation for fuzzy regression analysis, fundamentally resolving longstanding stability and reliability issues in parameter estimation. As anticipated, the convergence (12.3 ± 2.7 iterations) and stability of the algorithm conforms exactly to Banach’s fixed-point theorem. This theorem guarantees that contraction mappings in a complete metric space will decay exponentally in error. The geometric convergence illustrated in Figure 1 is an immediate result of the Lipschitz continuity of our operator T (L ≤ 0.82) which halted the propagation of error over the updated iterates. The ambiguity of the solution was reduced to a remarkable extent (18.3% narrower from the widths fo the parameters) than Tanaka’s method resulting from the uniqueness of the solution due to Banach’s constraints. While heuristic methods are free to explore multiple minimized fuzziness sets of parameters, the operator T, as a contraction mapping through Banach, compresses the solution space into a single, clearly defined attractor in parameter space. This quantifiable reduction in epistemic uncertainty in predicting was enabled by Banach’s assertions.

The reduction in prediction MSE of 25.1% was further evidence of the effectiveness of this approach. We formulated the regression problem within the d_∞-metric space of trapezoidal fuzzy numbers (TrFNs), while still retaining the structural relationships between variables via arithmetic operations ( Table 5). We did not introduce the distortions incurred by the use of linear programming forms by Tanaka or least squares forms by Diamond. For example, in the case of Tanaka, parameter widths will be inflated in order to incorporate outliers. LS-adopted forms often suffer from matrix ill-conditioning with improperly defined asymmetric uncertainties. In contrast, our gradient-based fixed-point iterations ( Equation 7), which iteratively tune the parameters, retain their geometric definitions, allowing for tighter and more accurate predictions intervals.

### Comparative analysis of prior work

The existential fragility of traditional fuzzy regression techniques is evident when systematically considered. Tanaka’s minimum fuzziness principle fails to guarantee solutions on multicollinear correlative cases, ^12^ noted; while in traditional LS-based approaches propagation of noise also results in unbounded error, because algebraic inversions do not consider topological constraints. Our approach avoids these issues by replacing deterministic optimization with a contractive mapping that converges uniquely (as proven) under certain conditions ( Theorem 2). The mathematics here underscores the statistically greater 4 × robustness to ±5 % input noise that we witness ( Table 8): where Tanaka’s and LS approaches are subject to error propagation, and unhelpful to degrees of freedom given that there is mathematical inherent failure, when the Lipschitz contraction condition L < 1 is met, perturbation to convergence is limited by default.

### Practical implications for engineering

These advancements have significant ramifications for uncertainty-aware modeling in material science and structural engineering. In high-stakes applications like concrete strength prediction, our method not only yields useful point estimates, but also estimate quantifiable uncertainty bounds based on the widths of the TrFNs (e.g., 32.7-37.3 MPa for a given 35 MPa specimen). This enables engineers to propagate imprecision through design calculations, supporting reliability-based decision-making. For instance, the 18.3% ambiguity reduction directly translates to narrower safety margins in load-bearing calculations, potentially reducing material overdesign by 12–15% while maintaining safety standards (ACI 318-19).

### Limitations and methodological considerations

Three limitations warrant emphasis. First, the contraction property hinges on the Lipschitz condition $K\cdot \max∥\tilde{X}∥<1$ , which may require data scaling for high-magnitude predictors (e.g., aggregate content exceeding 1,000 kg/m³). Second, while TrFNs efficiently model symmetric uncertainty, they may require extensions to Gaussian or LR-type fuzzy numbers for skewed distributions. Third, computational complexity scales linearly with predictors ( O(np) ), but for p>50 , the iterative gradient updates ( Equation 7) may benefit from quasi-Newton acceleration.

Critically, performance depends on appropriate fuzzification parameters ( Table 5). Overly narrow supports (e.g., $\delta_{2}<2\%$ ) artificially suppress uncertainty, while excessive widths inflate ambiguity. We recommend deriving $\delta_{1},\delta_{2}$ from domain-specific standards (e.g., ASTM tolerances) or bootstrap resampling. Future work should explore automated fuzzification via uncertainty quantification techniques like Monte Carlo dropout.

### Synthesis

This study demonstrates that fixed-point theory transcends theoretical elegance to deliver tangible improvements in fuzzy regression’s practicality. By embedding estimation within a complete metric space and leveraging Banach’s contractive principles, we resolve the existence-uniqueness-stability trilemma that has hindered the field since Tanaka’s pioneering work. The resulting framework bridges mathematical rigor with engineering utility—a critical step toward trustworthy uncertainty quantification in data-driven design.

## Conclusion

This study introduces a mathematically rigorous framework for fuzzy regression by employing fixed-point theory within the complete metric space of trapezoidal fuzzy numbers using the d∞-metric. Unlike classical approaches such as Tanaka’s minimum fuzziness or least-squares-based methods, which often lack guarantees of stability and uniqueness, our model ensures existence, uniqueness, and convergence through Banach’s Fixed-Point Theorem under verifiable Lipschitz conditions.

We developed a reliable iterative algorithm with a strict convergence criterion, implemented efficiently in Python. Empirical validation using the UCI Concrete Compressive Strength Dataset showed significant improvements in predictive accuracy (MSE reduction of 12.5%) and reduced uncertainty (18.3% decrease in parameter ambiguity) compared to benchmark methods. The model also demonstrated strong robustness under noise perturbation, making it practical for real-world engineering scenarios.

This work lays a solid mathematical foundation for fuzzy regression under uncertainty and supports its practical application in data-driven domains. Future research will explore extensions to non-trapezoidal fuzzy numbers (e.g., Gaussian, LR-shaped), nonlinear model structures, machine-learning-based fuzzification, and applications in economics and biomedical fields.

## Data Availability

All data supporting the findings of this study are openly available in Zenodo under a CC-BY 4.0 license. The dataset includes fuzzified trapezoidal data, numerical values underlying all tables and figures, simulation outputs, and extended materials. The complete dataset and extended data package can be accessed at: https://doi.org/10.5281/zenodo.17772389 (Abdalrahem, M. (2025).) ^3^
