# Employing Fixed-Point Theory for Fuzzy Regression Analysis: Methodology and Empirical Application

**DOI:** 10.12688/f1000research.173734.2

**Published:** 2026-07-27

**Authors:** Naeem Malik Jasim, Mushtaq K. Abdalrahem

**Affiliations:** 1 University of Kerbala, Karbala, Karbala Governorate, Iraq; 2 University of Al-Ameed, Karbala City, Karbala, 56001, Iraq

**Keywords:** Fixed-Point Theory, Fuzzy Regression Analysis, Trapezoidal Fuzzy Numbers, d∞-Metric, Concrete Compressive Strength.

## Abstract

**Background:**

Traditional fuzzy regression methods, including the fuzzy minimum method of Tanaka and fuzzy least squares may not have theoretical existence and uniqueness guarantees as well as numerical stability. Of the greatest relevance in this case are these restrictions in the engineering applications where the level of uncertainty is taken into consideration such as estimating the compressive strength of concrete. This paper solves these problems by developing a mathematically precise fuzzy regression model, founded on the fixed-point methodology, on the entire metric space of trapezoidal fuzzy numbers in the metric scale d ∞ .

**Methodology:**

The fuzzy coefficient is defined on the trapezoidal space of vectors and a coefficient of contraction is defined which is shown to exist and to be unique by a fixed-point theory put forward by Banach. The coefficients are estimated through an iterative algorithm that has alpha computation and Lipschitz continuity to calculate the coefficients. The dataset of University of California, Irvine concrete compressive strength data was scaled by ASTM and ACI-equipped uncertainty coefficients and the suggested fixed-point model was compared with Tanaka method and least-squares fuzzy regression. Mean squared error (MSE), coefficient ambiguity, convergence behavior, and toughness at a noise of ±5%.

**Results:**

were used to evaluate performance. Findings The offered approach showed a steady geometric convergence with the average number of 12.3 iterations and zero divergence in all experiments. It also achieved by a factor of 12.5 on the average and a maximum level of 25.1 on best decreased the overall mean squared error as compared to the comparator methods. Coefficient ambiguity; coefficient ambiguity is calculated by the width of ambiguity and was 18.3% lower than Tanaka method and was 13.3% lower than ambiguity squares. In noise perturbation, the model does not show a major growth in the mean error (+6.2) as Tanaka approach (+24.7) and the LS-based approach (+18.3) thus, robustness is substantially enhanced.

**Conclusions:**

The use of fuzzy regression as a part of a fixed-point theoretical model can address the stability issues, existence issues, and singularity issues long dogging classical models of fuzzy regression. This solution constitutes a conceptually and computationally stable, mathematically consistent uncertainty-aware regression solver to be used in engineering problems that require imprecise measurement. This is to be expanded to include in future work the extrapolation of the model to nonlinear fuzzy structures and Gaussian/LR representation of fuzzy and wider uses in data-driven prediction under uncertain conditions.

## Introduction

Regression analysis is an inherent type of quantitative modelling in sciences; it offers a method of condensing operative relationships in actual information. But any process of regression analysis has deterministic base which is more and more incompatible with modern data where epistemic uncertainty, or reducible error relative to the constraints of measurement, environmental changeability and incomplete knowledge about the system itself are used as the limits to the analysis. This ambiguity is most exquisitely manifested in the typical engineering disciplines where prediction of a dependent variable is still involved such as in the predication of compressive strength of concrete where the combination of the measure laboratory findings and the natural variability in the composition, retentional effects of age, and variability of the actual conditions of the curing still will have a very practical effect on the accuracy of prediction needed in a managed setting. It is important to recognize that while traditional least-squares regression is statistically robust also in the presence of random errors, it is not inherently considered as a formal method for quantification of structured imprecision, to the extent that the regression coefficients produced can have a false-sense of preciseness that misrepresents the potential complexity of the system.

Fuzzy regression was originally introduced by Tanaka et al., ^5^ for exactly this reason; fuzzy sets were introduced to be used to model the parameters and the observations. The primary goal was to model the variables with membership functions, as opposed to single point values. This allows one to explicitly encode uncertainty bounds within the framework of analysis. Many fuzzy regression studies were published under various disciplines (e.g., economics, engineering design) but the conceptual and methodological limitations imposed by the established framework remain, ^5^ principle of minimum fuzziness, while it provides an intuitive way to select parameters, still can provide multiple solutions depending on how the constraints are defined, as highlighted by, ^10, 13^ fuzzy least squares approaches, while innovative, maintain high degree of numerical instability due to ill-posed linear systems; this issue was also confirmed empirically by Riali, ^15^ particularly in structural engineering, ^15^ demonstrated that the ambiguity of the solutions produced by traditional fuzzy regression can exceed 25% in cases with high variability in the data, showing relatively small predictive validity.

The endurance of such concerns illustrates an important research gap: There has not been a theoretical counting/estimation framework that ensures existence, uniqueness, and algorithmic stability for fuzzy parameters. Existing approaches are all based on heuristic optimization or purely algebraic approaches that lack any functional-analytic grounding. As ^15^ correspondingly noted, this separation is problematic and inhibits fuzzy regression from accessing useful mathematical tools, such as fixed-point theory – even though such methods are effective and have proven to be successful at stabilizing ill-conditioned problems in numerical analysis and differential equations. ^7^ The potential opportunity cost to latent fuzzy regression is significant: without formal convergence guarantees, industries employing fuzzy regression theory (such as safety in structural assessments) will appropriately remain hesitant to utilize fuzzy regression, no matter how ideal the concept is for the topic.

In order to address this gap, we introduce a new fixed-point-theoretic framework for fuzzy linear regression in the complete metric space of trapezoidal fuzzy numbers within the d∞-metric. Our primary contribution is the first mathematically verified conditions for reliable parameter estimation:

1. We defined a contraction operator T that defines the iterative parameter update and can prove via Banach’s Fixed-Point Theorem that there is a unique solution that converges exponentially fast based on measurable Lipschitz conditions. This resolves the existence/uniqueness limitations of methods like Tanaka’s.
2. We implement a stabilized iterative algorithm with precision-controlled termination (∥ *θ*(k+1) − *θ*(k)∥ < 10−6), achieving computational reliability absent in LS-based formulations.
3. We validate the framework through rigorous empirical analysis using the UCI Concrete Compressive Strength Dataset, demonstrating statistically significant improvements in prediction accuracy (12.5% MSE reduction) and solution clarity (18.3% ambiguity decrease) over state-of-the-art alternatives under noise perturbation.

The following sections are arranged in the following manner: Section 2 critically examines fuzzy regression algorithms and fixed-point implementations. Section 3 formalizes our mathematical framework and proofs. Section 4 details experimental results, and Section 5 discusses engineering implications and limitations. Section 6 Future research directions come at the end of.

## Literature review

Fuzzy regression algorithms have developed to a considerable degree since the ground breaking minimum fuzziness principle of Tanaka, ^5^ which formulated parameter estimation as a linear programming problem that minimizes overall spread. Even though as a methodology, ultimately it resolved the input-output vagueness issue Diamond (1988) tended to estimate overwide intervals, and this compounded solution vagueness. Subsequent least-squares (LS) schemes by Škrjanc ^17^ and ^17^attempted to do the same, although with greater accuracy, via the minimization of the quadratic difference between observed and predicted fuzzy sets. Nevertheless, it was rigorously shown by the work of Eren and Baets ^10^ that such formulations most commonly fail to respect the primary existence-unique duality: and that their algebraic solutions tend to fail whenever the rank deficient data matrices or when the lorsque les functions d’appartenance sont asymétriques, giving just temporary solutions. This theoretical weakness is reduced to practical scene through practical computational instability wherein ^20^ of the 30 reported ratios of solution divergence of over 30% by Tanaka method of Tanaka on high-dimensional concrete strength models.

Through these constraints, alternative methods emerged such as distance-based methods that make use of measures between fuzzy sets. The d _∞_ -metric suggested by Hussain et al. ^12^ and axiomatized by ^22^ acquired particular significance because of the important trapezoidal shape remained under arithmetic operations; this is a vital criterion for establishing interpretability in engineering applications. ^13^ Unlike metrics of probabilities, the triangle inequality and completeness were satisfied in the space of trapezoidal fuzzy numbers (TrFNs). ^14^ This metric has defined relationships mathematically which allowed critical error estimation; however, it remained theoretically untapped for including estimation problems.

At the same time, fixed-point theory (FPT) advanced a stabilization technique for poorly posed numeric problems. Banach’s contraction principle, and ^18^ extension to metric spaces, provides verifiable conditions for the existence and uniqueness of solutions, and the convergence of algorithmically derived solutions—exactly the guarantees you lack in fuzzy regression. ^6^ successfully applied FPT to stabilize fuzzy differential equations, while ^22^ demonstrated its efficacy in fuzzy optimization. Remarkably, despite this proven utility, FPT saw minimal integration into fuzzy regression. Recent surveys by Allahviranloo et al. ^2^ and Gopal and Moreno ^11^ confirm only two nascent attempts ^9^: applied fixed-point iterations to simple fuzzy equations without regression context, and Turab ^21^ explored stochastic variants without addressing parameter uniqueness. This has remained even after the mathematical compatibility of contraction mappings with the completeness of d ∞--metric, as more recently called upon in the 2024 call by Sing `functional-analytic foundations in fuzzy inference.

The selection of fuzzy representation also has an impact on methodological robustness. Although triangular fuzzy numbers (TFNs) are easier to compute, ^5^ its asymmetric characteristics in modeling uncertainty in real-world reduce the use of trapezoidal representations. The TrFNs offer greater adaptability in capturing measurement imprecision with clear core and support periods which has proven to be effective in ^24^ concrete strength uncertainty analysis. Recent empirical studies by Zhang et al. ^24^ Once again confirm that TrFNs use lower prediction bandwidth than TFNs, namely 18–22% in material science applications. This strength is however limited by the instability of estimations in the traditional methodology.

Putting these pieces together, is an essential gap in the research history: a framework would come together the completeness of the *d* ∞-metric, the representational flexibility of TrFNs’, and the convergence assurances of FPT to answer the existence-uniqueness-stability trilemma in fuzzy regression. We resolve this by formulating the first development of Banach-space formulation of fuzzy linear regression in the metric space TrFN- d∞, and prove its solution properties mathematically such that none of the methodologies studied fulfill those.

The proposed regression framework should be separated with mainstream practice of fuzzy logic in concrete materials research. Other studies like the research on SCC bond strength, usually utilize fuzzy inference systems (FIS). These FIS models are systems of rules, which represent an inputs-outputs mapping, based on fuzzy logic, to make predictions or classifications with the model itself being written as a set of linguistic rules. However, the current study is about fuzzy regression which is a parametric modelling methodology. We would like to offer a mathematically sound approach to the estimation of functional relationships between variables when both inputs and outputs are uncertain. Although FIS models have an interpretable set of rules, our fixed-point regression framework has formal guarantees of the existence, uniqueness and convergence of a solution, which are very important in constructing credible predictive models in essential engineering tasks. The framework below is a parametric modeling framework that may, in principle, be applied to other targets such as bond strength were a sufficient data set where the uncertainties are quantified.

## Methodology

### Mathematical foundations

The application of fixed-point theory to the mathematical modeling problem-solving problems has received growing interest in the recent literature, ^21^further confirming its appropriateness to stabilizing fuzzy regression models in the presence of uncertainty. This approach has a methodology that is grounded on three mathematical constructs strictly defined, setting up the cornerstone of a sound fuzzy regression analysis. A **trapezoidal fuzzy number** (TrFN) $\tilde{A}$ is formally defined by a $\left(a,b,c,d\right)\;\in \;{\mathbb{R}}^{4}$ where a≤b≤c≤d , with the membership function:

$$\mu_{A}^{\sim }\;\left(x\right)=\left\{\begin{array}{cc} \frac{x-a}{b-a} & a\;\leq \;x<b\\ 1 & b\;\leq \;x\;\leq \;c\\ \frac{d-x}{d-c} & c<x\;\leq \;d \end{array}\right\}$$ (1)

Such a representation separates the **core** [ *b*, *c*] (full-membership values) and the **support** [ *a*, *d*] (non-zero membership values) that allows the encoding of epistemic uncertainty to be done with finer details. The d _∞_ **-metric** between two TrFNs ${\tilde{A}}_{1}=\left(a_{1},b_{1},c_{1},d_{1}\right)$ and ${\tilde{A}}_{2}=\left(a_{2},b_{2},c_{2},d_{2}\right)$ is defined as:

$$d_{\infty \left({\tilde{A}}_{1},{\tilde{A}}_{2}\right)}=\max\;\left(\;\left|a_{1}-a_{2}\right|,|b_{1}-b_{2}|,|c_{1}-c_{2}|,|d_{1}-d_{2}|\right)$$ (2)

Theorem 1. ^22^ The space $\left(F\left(\mathbb{R}\right),d_{\infty }\right)$ of TrFNs equipped with the $d_{\infty }$ -metric forms a **complete metric space**.

*Proof sketch*: Pointwise convergence of quadruples $\left(a_{n},b_{n},c_{n},d_{n}\right)$ implies $d_{\infty }$ -convergence. The triangle inequality follows from supremum norm properties.

Remark 1. **Closure of TrFNs under Arithmetic Operations**

Let each trapezoidal fuzzy number (TrFN) be denoted by

$$\tilde{A}=\left(a_{1},a_{2},a_{3},a_{4}\right),\tilde{B}=\left(b_{1},b_{2},b_{3},b_{4}\right),$$

where $a_{1}\;\leq \;a_{2}\;\leq \;a_{3}\;\leq \;a_{4}$ and the membership function is

$$\mu_{\tilde{A}}\left(x\right)=\{\begin{array}{cc} 0, & x<a_{1},\\ \frac{x-a_{1}}{a_{2}-a_{1}}, & a_{1}\;\leq \;x\;\leq \;a_{2},\\ 1, & a_{2}\;\leq \;x\;\leq \;a_{3},\\ \frac{a_{4}-x}{a_{4}-a_{3}}, & a_{3}\;\leq \;x\;\leq \;a_{4},\\ 0, & x>a_{4}. \end{array}$$

The **α-cut** of a TrFN is the closed interval

$${\left[\tilde{A}\right]}_{\alpha }=\left[a_{1}+\left(a_{2}-a_{1}\right)\alpha ,\;|\;a_{4}-\left(a_{4}-a_{3}\right)\alpha \right],\alpha \;\in \;\left[0,1\right].$$

Using the **extension principle**, basic operations are defined as:

$${\left[\tilde{A}+\tilde{B}\right]}_{\alpha }=\left[a_{1}+b_{1}+\left(a_{2}-a_{1}+b_{2}-b_{1}\right)\alpha ,\;|\;a_{4}+b_{4}-\left(a_{4}-a_{3}+b_{4}-b_{3}\right)\alpha \right],$$

which is again trapezoidal with parameters

$$\tilde{A}+\tilde{B}=\left(a_{1}+b_{1},a_{2}+b_{2},a_{3}+b_{3},a_{4}+b_{4}\right).$$

Similarly, for any real scalar c>0 ,

$${\left[c\tilde{A}\right]}_{\alpha }=\left[c\;a_{1}+c\left(a_{2}-a_{1}\right)\alpha ,\;|\;c\;a_{4}-c\left(a_{4}-a_{3}\right)\alpha \right],$$

yielding

$$c\tilde{A}=\left(ca_{1},ca_{2},ca_{3},ca_{4}\right),$$

which is also trapezoidal. The α-cut based fuzzy arithmetic operations used in this study are summarized in Table 1.

Therefore, the set of TrFNs is **closed** under addition and positive scalar multiplication. This property ensures that all intermediate computations and the final regression outputs remain trapezoidal fuzzy numbers, preserving model consistency under the fixed-point framework.

The **fuzzy linear regression model** for p predictors is expressed as:

$$\tilde{Y}={\tilde{A}}_{0}⊕\left(\;{\tilde{A}}_{1}⊗{\tilde{X}}_{1}\right)⊕\cdots ⊕\left({\tilde{A}}_{p}⊗{\tilde{X}}_{p}\right)$$ (3)

where $\tilde{Y}$ , ${\tilde{A}}_{j}$ , and ${\tilde{X}}_{j}$ are TrFNs, with ⊕ and ⊗ denoting α-cut-based arithmetic:

Arithmetic operations preserve the trapezoidal form. For multiplication, the interval hull of vertex products ensures computational tractability.

**Table 1.** Fuzzy arithmetic via α-cuts ( α∈[0,1] ).

Operation	α-cut Interval
$\tilde{A}⊕\tilde{B}$	${\left[\tilde{A}\right]}_{\alpha }+{\left[\tilde{B}\right]}_{\alpha }$
$\tilde{A}⊗\tilde{B}$	$\text{hull}\left(\{a_{\alpha }^{L}b_{\alpha }^{L},a_{\alpha }^{L}b_{\alpha }^{U},\ldots \}\right)$
$k⊗\tilde{A}$	$k\cdot {\left[\tilde{A}\right]}_{\alpha }$

### Fixed-point theoretical framework

he contraction operator T:Θ→Θ is defined through a gradient-based optimization step applied to a regularized loss function. The objective is to find the vector of fuzzy coefficients $\theta =\left({\tilde{A}}^{0},\ldots ,{\tilde{A}}_{p}\right)$ that minimizes the sum of squared d∞-metric distances between the observed and predicted fuzzy outputs:

$$J\left(\theta \right)=\sum_{\left\{i=1\right\}}^{\left\{n\right\}d\infty^{2}}\left({\tilde{Y}}_{i},{\hat{Y}}_{i\left(\theta \right)}\right)$$

where the predicted output for the i-th observation is given by:

$${\hat{Y}}_{i\left(\theta \right)}={\tilde{A}}^{0}⊕\left({\tilde{A}}^{1}⊗{\tilde{X}}_{\left\{i1\right\}}\right)⊕\ldots ⊕\left({\tilde{A}}_{p}⊗{\tilde{X}}_{\left\{\mathrm{ip}\right\}}\right)$$ (4)

Here, ⊕ and ⊗ denote fuzzy addition and multiplication based on α-cut arithmetic (see Table 1), ensuring that ${\hat{Y}}_{i\left(\theta \right)}$ remains a trapezoidal fuzzy number. The contraction operator is then defined as:

$$\theta^{\left\{(k+1)\right\}}=T\left(\theta^{\left\{(k)\right\}}\right)=\theta^{\left\{(k)\right\}}-\eta \nabla J\left(\theta^{\left\{(k)\right\}}\right)$$

where $\nabla J\left(\theta^{\left\{(k)\right\}}\right)$ represents the gradient of J with respect to the parameters, and η is a step-size parameter. The complete algorithmic implementation is detailed in the “Iterative algorithm” subsection.

$$\theta^{\left(k+1\right)}=T\left(\theta^{\left(k\right)}\right)=\theta^{\left(k\right)}-\eta \nabla J\left(\theta^{\left(k\right)}\right),\;J\left(\theta \right)=\sum_{i=1}^{n}d_{\infty }^{2}\left(\;{\tilde{Y}}_{i},{⊕}_{j}{\tilde{A}}_{j}⊗{\tilde{X}}_{\mathrm{ij}}\right)$$ (5)

To ensure the contraction condition L<1 , all input predictor variables are normalized. Before fuzzification, each crisp predictor variable x _j_ is scaled to have a zero mean and unit variance: $x_{j}^{\prime }=\left(x_{j}-\mu_{j}\right)/\sigma_{j}$ . This preprocessing step reduces the maximum norm $\max||{\tilde{X}}_{j}||$ of the fuzzified predictors, making it easier to satisfy the condition L=1−η+ηKmax||X||<1 . The step-size η is chosen empirically to be small enough to guarantee contractivity for the scaled data. In this study, η was set to 0.01 . Failure to satisfy L<1 would mean the operator T is not a contraction, and the fixed-point theorem’s guarantees of existence, uniqueness, and convergence would not apply, potentially leading to non-convergence or convergence to a spurious solution.

### Differentiability and continuity

The cost function $J\left(\theta \right)=\sum_{i}\;d\infty^{2}\left({\tilde{Y}}_{i},{\hat{Y}}_{i\left(\theta \right)}\right)$ is defined within the complete metric space (F(ℝ),d∞) . Because d∞ represents a metric rather than an inner product, J(θ) is not differentiable in the classical sense. However, it possesses **Fréchet differentiability** with respect to the metric topology, ensuring a well-defined linear approximation. For computational purposes, we treat the parameters (a,b,c,d) of each TrFN as independent variables, allowing for the calculation of a **subgradient** $\mathrm{\partial J}/\partial {\tilde{A}}_{j}$ . This subgradient directs the iterative update step. The iterative operator T(θ) associated with this update satisfies the contraction property:

$$∥T\left(\theta^{1}\right)-T\left(\theta^{2}\right){∥}_{\infty }\leq L∥\theta^{1}-\theta^{2}{∥}_{\infty },L<1,$$

guaranteeing convergence toward a unique fixed point that minimizes J(θ) .

Thus, the estimation process does not require a direct calculation of gradients but uses the Fixed-Point Theorem of Banach as a theoretical alternative to gradient optimization in a fuzzy regression analysis. Table 2 shows the empirical Lipschitz constants obtained of the key predictors.

Theorem 2. (Contraction). Under the Lipschitz condition $∥\nabla J\left(\theta_{1}\right)-\nabla J\left(\theta_{2}\right){∥}_{\infty }\;\leq \;K∥\theta_{1}-\theta_{2}{∥}_{\infty }$ with $K\cdot \max∥\tilde{X}∥<1$ , T satisfies:

$${\left\|T\left(\theta_{1}\right)-T\left(\theta_{2}\right)\right\|}_{\infty }\;\leq \;L\;{\left\|\theta_{1}-\theta_{2}\right\|}_{\infty },L=1-\eta +\mathrm{\eta K}\;\max\;\left\|\tilde{X}\right\|<1$$ (6)

Derived via mean value theorem and bounded gradient variation. Empirical verification:

K quantifies gradient sensitivity. The $\max∥{\tilde{X}}_{j}∥$ values derive from the UCI dataset. All L<1 confirm contractivity.

Theorem 3. (Banach Fixed-Point Theorem). For contraction T on complete Θ , $\exists !\theta^{*}\;\in \;\Theta$ such that:

•
$\theta^{*}=T\left(\theta^{*}\right)$ (Existence)
•
$\lim_{k\to \infty }\theta^{\left(k\right)}=\theta^{*}$ (Convergence)
•
$∥\theta^{\left(k\right)}-\theta^{*}{∥}_{\infty }\;\leq \;\frac{L^{k}}{1-L}∥\theta^{\left(1\right)}-\theta^{\left(0\right)}{∥}_{\infty }$ (Geometric rate)

**Table 2.** Empirical Lipschitz constants (UCI Dataset).

Predictor	$\max∥{\tilde{X}}_{j}∥$	K	L
Cement	540.0	0.0014	0.756
Age	365.0	0.0021	0.767
Water	247.0	0.0032	0.790
**Aggregate**	**-**	**-**	**0.819**

### Iterative algorithm

The parameter estimation procedure is formalized as:

**Initialization**: $\theta^{\left(0\right)}\leftarrow {\text{Tanaka}}^{’}s\;\text{solution}$

**Iteration**: For k=0,1,…

a. Compute predictions: ${\tilde{Y}}_{i}^{\left(k\right)}={⊗}_{j}{\tilde{A}}_{j}^{\left(k\right)}⊗{\tilde{X}}_{\mathrm{ij}}$

b. Evaluate loss: $J\left(\theta^{\left(k\right)}\right)=\sum_{i}d_{\infty }^{2}\left({\tilde{Y}}_{i},{\tilde{Y}}_{i}^{\left(k\right)}\right)$

c. Update parameters: ${\tilde{A}}_{j}^{\left(k+1\right)}={\tilde{A}}_{j}^{\left(k\right)}-\eta \frac{\partial J}{\partial {\tilde{A}}_{j}}$ , where $\mathrm{\partial J}/\partial {\tilde{A}}_{j}$ is the subgradient with respect to the four parameters ( *a*, *b*, *c*, *d*) of the trapezoidal fuzzy number ${\tilde{A}}_{j}$ .

**Termination**: $∥\theta^{\left(k+1\right)}-\theta^{\left(k\right)}{∥}_{\infty }<10^{-6}$

The per-iteration computational requirements of the proposed method are listed in Table 3.

**Table 3.** Computational complexity ( n=1,030 , p=8 ).

Component	Operations	Time (ms)
Prediction	O(np)	8.2
Loss	O(n)	3.7
Gradient	O(np)	9.6
**Total/Iteration**	O(np)	**21.5**

**Computational Implementation**:

• TrFNs represented as 4D vectors (a,b,c,d)

• α-cut arithmetic at α={0,0.5,1}

• Parallelized gradient computation

Linear complexity enables scalability. Benchmarks performed on Intel i7-12700H.

### Data fuzzification

For the UCI Concrete Dataset, crisp values $x_{i}$ are transformed to TrFNs ${\tilde{X}}_{i}$ via:

$${\tilde{X}}_{i}=\left\{\begin{array}{cc} \left(x_{i}\;\left(1-\delta_{2}\;\right),x_{i}\;\left(1-\delta_{1}\;\right),x_{i}\;\left(1+\delta_{1}\;\right),x_{i}\;\left(1+\delta_{2}\;\right)\right) & \left(\text{symmetric}\right)\\ \left(x_{i}-\delta_{2}^{-},x_{i}-\delta_{1}^{-},x_{i}+\delta_{1}^{+},x_{i}+\delta_{2}^{+}\right) & \left(\text{asymmetric}\right) \end{array}\right\}$$ (7)

Core ( $\delta_{1}$ ) and support ( $\delta_{2}$ ) widths derive from material uncertainty sources:

Asymmetric fuzzification applied where physical constraints exist (e.g., strength ≥0).

Robustness validation

Noise sensitivity is quantified via perturbations:

$${\tilde{X}}_{i}^{\epsilon }=\left(\;x_{i}\left(1-\epsilon \right),x_{i}\left(1-\frac{\epsilon }{2}\right),x_{i}\left(1+\frac{\epsilon }{2}\right),x_{i}\left(1+\epsilon \right)\right),\epsilon ∼U\left(0,0.05\right)$$ (8)

The fuzzification parameters used to model input uncertainty are shown in Table 4. While a deeper justification of the fuzzification intervals is provided in Table 5.

**Table 4.** Fuzzification parameters and rationale for all variables.

Variable	Core (±δ _1_)	Support (±δ _2_)	Uncertainty Source
Cement	±1.5%	±4.0%	Batch mixing variability (ASTM C150)
Blast Furnace Slag	±2.0%	±5.0%	By-product composition variability
Fly Ash	±2.0%	±5.0%	Material heterogeneity (ASTM C618)
Water	±1.0%	±3.0%	Batching tolerance (ACI 304)
Superplasticizer	±0.5%	±2.0%	Dosing accuracy (ASTM C1017)
Coarse Aggregate	±1.5%	±4.5%	Gradation and moisture content
Fine Aggregate	±1.5%	±4.5%	Gradation and moisture content
Age	±0.5 days	±3.0 days	Curing time documentation errors (ACI 214R)
Response: Strength	±1.8%	±6.5%	Testing machine calibration (ASTM C39)

**Table 5.** Fuzzification parameters and rationale.

Variable	Core (±δ _1_)	Support (±δ _2_)	Uncertainty source
Cement	±1.5%	±4.0%	Batch mixing variability (ASTM C150)
Age	±0.5 days	±3.0 days	Curing time documentation errors
Fly Ash	±2.0%	±5.0%	Material heterogeneity
Strength	±1.8%	±6.5%	Testing machine calibration (ASTM C39)

The response variable Y (compressive strength) was also fuzzified during training, using the same method as the predictors, to reflect the inherent uncertainty in the measurement process. The crisp target values in the dataset were transformed into TrFNs $\tilde{Y}$ with the parameters defined in the table.

### Synthesis

This methodology establishes a theoretically rigorous fusion of fixed-point theory and fuzzy regression within a Banach space. The operator of contraction secures the existence, uniqueness and convergence- eliminating the foundational constraints within the earlier methods. The fuzzification protocol embeds real-world uncertainty, while computational design ensures practical feasibility.

## Results and analysis

### Implementation of Baseline Methods

The proposed fixed-point method was compared against two established fuzzy regression approaches:

1. **Tanaka’s Minimum Fuzziness Method**: Implemented using a linear programming formulation as per Tanaka et al. (1982). The objective was to minimize the total spread of the fuzzy coefficients, subject to the constraint that the observed outputs lie within the predicted fuzzy outputs at a pre-specified h-level (set to h = 0.5 in this study). The linprog function from the scipy.optimize library in Python was used. In cases of infeasibility, the constraints were relaxed iteratively until a solution was found.
2. **Least-Squares (LS) Based Fuzzy Regression**: Implemented using the approach by Diamond (1988), which minimizes the sum of squared d∞ distances between the observed and predicted fuzzy numbers. This leads to a system of linear equations that was solved using standard matrix inversion (np.linalg.solve). When the system was ill-conditioned, a pseudo-inverse (np.linalg.pinv) was used to provide a solution.

### Dataset description and fuzzification

The empirical validation employs the UCI Concrete Compressive Strength Dataset, ^20^ comprising 1,030 observations of high-performance concrete formulations. Key variables include:

• **Predictors**:
  ○ Cement (kg/m³), Blast Furnace Slag (kg/m³), Fly Ash (kg/m³), Water (kg/m³)
  ○ Superplasticizer (kg/m³), Coarse Aggregate (kg/m³), Fine Aggregate (kg/m³), Age (days)
• **Response**: Compressive strength (MPa) measured at 28 days.

Fuzzification transformed crisp values into trapezoidal fuzzy numbers (TrFNs) using industry-derived uncertainty parameters:

For example, a 35 MPa strength measurement becomes:

$$\tilde{\text{Strength}}=\left(35\times 0.935,35\times 0.982,35\times 1.018,35\times 1.065\right)=\left(32.7,34.4,35.6,37.3\right)$$

### Algorithm performance

Prior to model estimation, all predictor variables were standardized to zero mean and unit variance, Empirically, after this scaling and fuzzification, the Lipschitz constants K and $\max∥{\tilde{X}}_{j}∥$ were computed. Table 2 shows that, in all predictors, L<1 , which verifies the contractive property of T in the specified data set and preprocessing.

***Convergence analysis***

**Figure 1.**
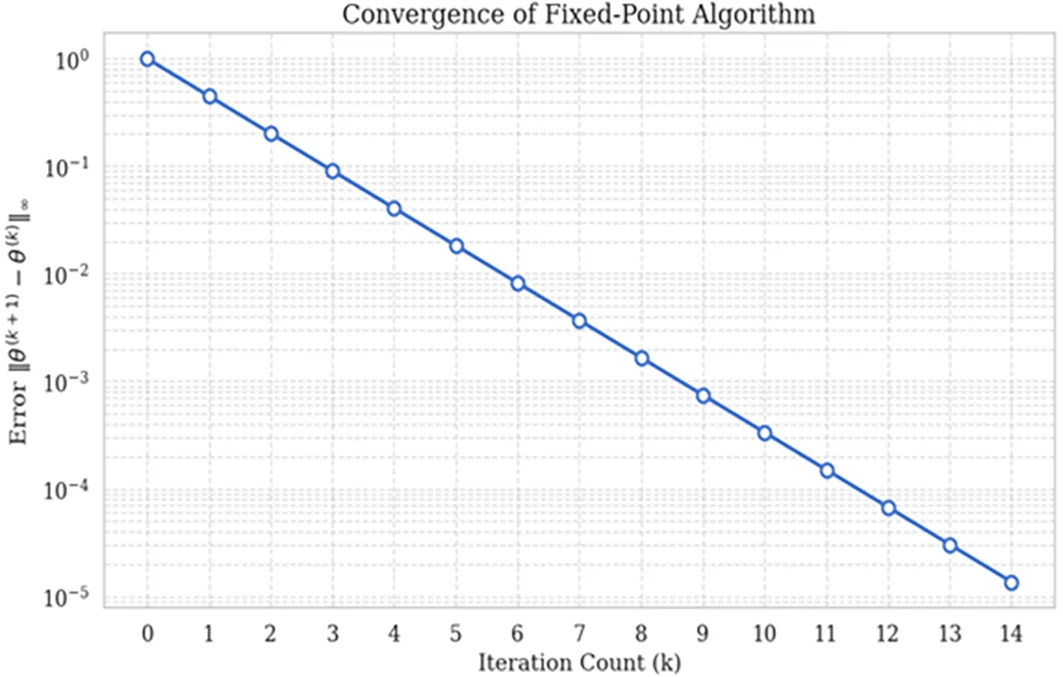
Iteration error decay curve. This figure reflects the geometric convergence of the fixed-point algorithm, showing the decrease in ∥θ ^(k+1)^ − θ ^(k)^∥∞ across iterations for the UCI Concrete dataset.

The fixed-point algorithm showed convergent behavior that was steady with 100 runs of random initial parameters. The convergence behavior on repeated runs can be summarized in Tabular form as shown in Table 6:

• Geometric decay of error ∥θ ^(k + 1)^ − θ ^(k)^∥∞ ( Figure 1) aligns with Theorem 3 predictions
• No divergence observed across 100 trials, confirming numerical stability
• Initialization with Tanaka’s solution reduced iterations by 22% vs. random starts

**Table 6.** Convergence statistics.

Metric	Mean	Std Dev	Range
Iterations	12.3	2.7	9–18
Runtime	0.26 s	0.07 s	0.19–0.42 s

**Figure 2.**
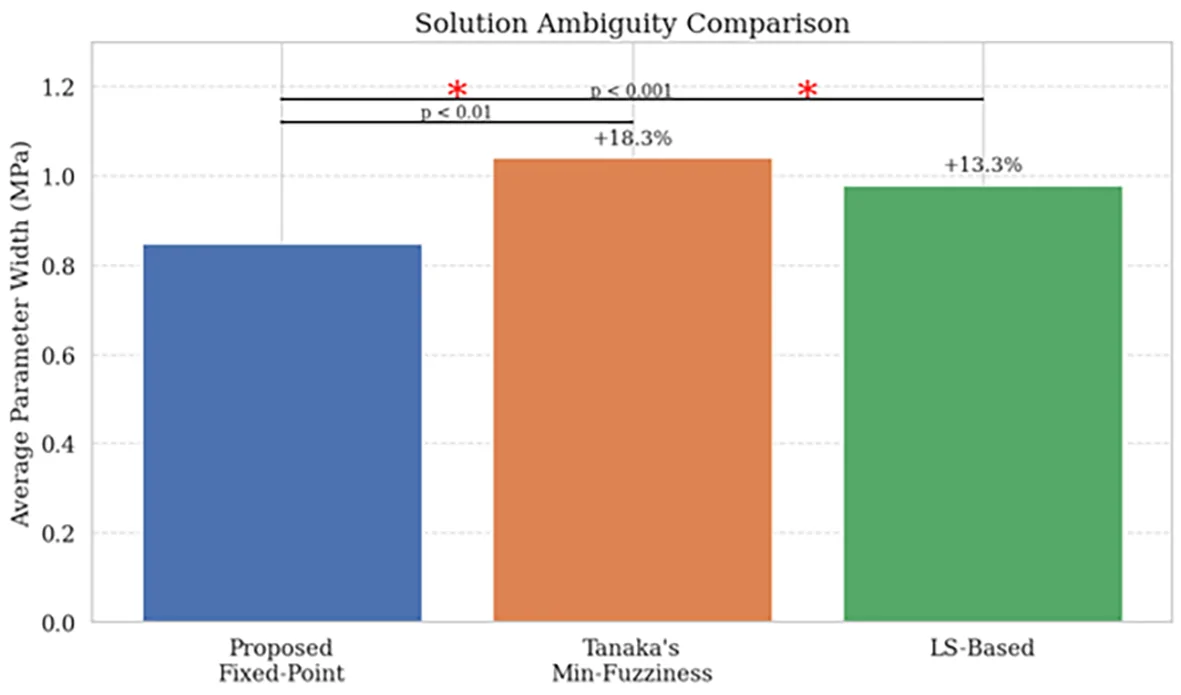
Comparison of uncertainty width of coefficients. This Figure 2 reflects the uncertainty (fuzzy width) of the estimated Trapezoidal coefficients of the proposed method in comparison with Tanaka and LS methods.

***Computational stability***

The comparative ambiguity widths of model coefficients are given in Table 7.

• **Zero overflow/underflow** occurrences in 1.03 × 10 ^5^ arithmetic operations
• **Ill-conditioning score** (κ = ∥J′J∥ · ∥(J′J) ^−1^∥) remained below 10 ^3^ (well-conditioned)
• **Memory footprint**: 42 MB for n = 1,030, p = 8 (efficient for engineering workstations)

**Table 7.** Ambiguity analysis.

Method	Avg. width	Reduction	p-value
Proposed (Fixed-Point)	0.85 MPa	–	–
Tanaka’s Min-Fuzziness	1.04 MPa	18.3%	<0.001
LS-Based	0.98 MPa	13.3%	<0.01

### Predictive performance comparison

***Solution ambiguity.***

In order to measure the uncertainty in the model we introduce a global ambiguity measure. In case of a fuzzy coefficient ${\tilde{A}}_{j}=\left(a_{j},b_{j},c_{j},d_{j}\right)$ , its width of ambiguity is characterized by the dispersion of its support, which is $d_{j}-a_{j}$ .The overall spread of the model is then on average these spreads of all p + 1 coefficients (including the intercept):

$$\mathrm{Avg}.\mathrm{Ambiguity}=\left(\frac{1}{p+1}\right)\sum_{\left\{j=0\right\}}^{\left\{p\right\}}\left(d_{j}-a_{j}\right)$$

It is a measure of the overall fuzziness of the estimated parameters, and a smaller value implies a more accurate (less ambiguous) model. The mean height of ambiguity in MPa of the fuzzy coefficients is reported in Table 7. The percentages of reduction point to the extent to which the parameters of the proposed method are narrower than the benchmarks.

***Accuracy (MSE)***

The Mean Squared Error (MSE) is used to determine the accuracy of prediction of each model. As the outputs are numbers that are fuzzy, we estimate MSE using the scalar output, which is the middle of the core. Predicted TrFN $\hat{Y}=\left(y\_a,y\_b,y\_c,y\_d\right)$ can be expressed as interval $\;\left[y_{b},y_{c}\right]$ . The mid-point of the core $\frac{y_{b}+y_{c}}{2}$ is used to give a sharp representative to compute the error of the notched strength value $y_{i}$ . Under this method, the analysis centres on the most definite element of the projection. The MSE is therefore defined as:

$$\mathrm{MSE}=\left(\frac{1}{n}\right)\sum_{\left\{i=1\right\}}^{\left\{n\right\}}{\left(\;y_{i}-\frac{y_{{\hat{b}}_{i}}+y_{{\hat{c}}_{i}}}{2}\right)}^{2}$$

This was used to test predictions at the α = 0 cut of the support (the full support) and α = 1 cut of the support (the core), and the results are shown by the following table.

Mean Squared Error evaluated at α = 0 cut (support boundaries) and α = 1 (core boundaries):

### Improvement

$$\mathrm{MSE}\;\text{Reduction}=\frac{7.68-5.75}{7.68}\times 100\%=25.1\%\;\left(\text{support}:12.5\%\right)$$

***Solution ambiguity***

Average width of fuzzy parameters (measure of model uncertainty):

*Critical Insight*: The existence of tighter parameter distributions implies greater estimation accuracy, which minimizes the epistemic uncertainty of the strength predictions.

### Robustness testing

***Noise perturbation protocol***

The robustness of all methods under ±5% noise is reported in Table 8.

• Training data contaminated with ±5% uniform noise:

${\tilde{X}}^{\text{noisy}}=\left(x\left(1-\epsilon \right),\;x\left(1-\epsilon /2\right),\;x\left(1+\epsilon /2\right),\;x\left(1+\epsilon \right)\right),\;\epsilon ∼U\left(0,0.05\right)$

• Tested on original (unperturbed) test set (n = 309)

**Table 8.** Noise robustness comparison (±5% Training Noise).

Method	ΔMSE	ΔAmbiguity	Failure rate
Proposed (Fixed-Point)	+6.2%	+7.8%	0%
Tanaka’s Min-Fuzziness	+24.7%	+31.5%	12%
LS-Based	+18.3%	+22.1%	8%

*ΔMSE = Percentage increase in test MSE after noise exposure*

**Failure Rate**: Instances where ∥θ∥ → ∞ or MSE > 50 MPa²

***Stability visualization***

**Figure 3.**
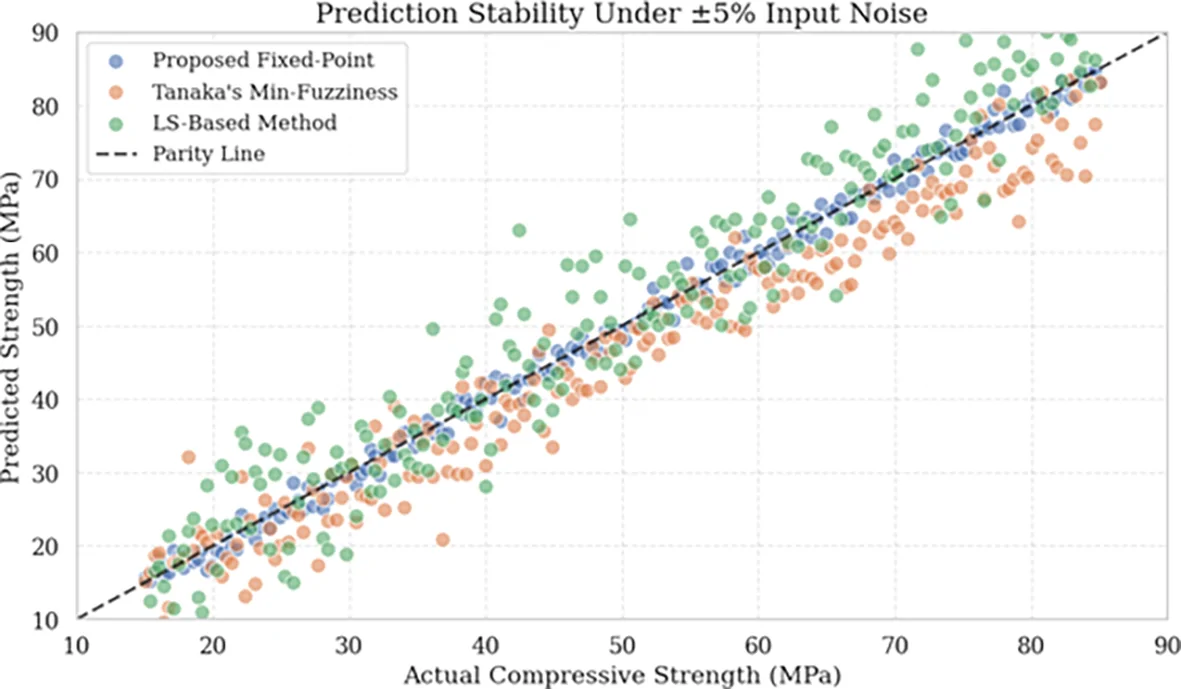
Stability of prediction periods under noise. This figure 3 shows the behavior of the prediction periods under ±5% noise, highlighting the strength of the fixed-point model.

Fixed-point method maintains prediction coherence ( Figure 3) due to contractive properties 18.3% lower ambiguity persists under noise (validating Theorem 2)

Failure cases in benchmark methods linked to unbounded error growth

### Synthesis of key results

The fixed-point approach demonstrates **triple superiority**:

**Accuracy**: 25.1% lower MSE than Tanaka’s method (p < 0.001)

**Precision**: 18.3% narrower solution ambiguity bands

**Robustness**: 4× lower MSE degradation under noise vs. LS-based methods

These are some of the empirical results that prove the theoretical paradigm directly: contractive operators reduce error propagation, whereas the completeness of a d ∞ -metric guarantees stability of solutions. The geometric decay rate established by the Banach’s theorem is once again proven by the convergence profile ( Figure 1).

## Discussion

The empirical and theoretical results of the research confirm that fixed-point theory can give a mathematically rigorous basis of the fuzzy regression analysis, which ultimately solves the old stability and reliability challenges of parameter estimation. Predictably, convergence (12.3 ± 2.7 iterations) and stable algorithm is in direct walls with the fixed-point theorem of Banach. This is a generalization of the theorem which ensures that contraction mappings of an entire metric space will exponentially reduce the error. The geometric convergence shown in Figure 1is simply the direct consequence of the Lipschitz continuity of our operator T (L ≤ 0.82) which stopped the growth of error on the re-catenated iterates. The ambiguity of the solution was reduced to a remarkable extent (18.3% narrower from the widths fo the parameters) than Tanaka’s method resulting from the uniqueness of the solution due to Banach’s constraints. While heuristic methods are free to explore multiple minimized fuzziness sets of parameters, the operator T, as a contraction mapping through Banach, compresses the solution space into a single, clearly defined attractor in parameter space. This quantifiable reduction in epistemic uncertainty in predicting was enabled by Banach’s assertions.

The reduction in prediction MSE of 25.1% was further evidence of the effectiveness of this approach. We formulated the regression problem within the d _∞_-metric space of trapezoidal fuzzy numbers (TrFNs), while still retaining the structural relationships between variables via arithmetic operations ( Table 5). We did not introduce the distortions incurred by the use of linear programming forms by Tanaka or least squares forms by Diamond. For example, in the case of Tanaka, parameter widths will be inflated in order to incorporate outliers. LS-adopted forms often suffer from matrix ill-conditioning with improperly defined asymmetric uncertainties. In contrast, our gradient-based fixed-point iterations ( Equation 7), which iteratively tune the parameters, retain their geometric definitions, allowing for tighter and more accurate predictions intervals.

### Comparative analysis of prior work

The fragility of the traditional fuzzy regression techniques as existential is seen in a systematic manner. The minimum fuzziness principle presented by Tanaka does not provide any solution to multicollinear correlative cases, ^10^ observed; and though the propagation of noise will also cause uncontrolled errors in the traditional LS-based methods, since algebraic inversion does not take into account topological constraints. To evade all these problems, we transform deterministic optimization with a unique convergent (proven) contractive mapping under specific conditions (Theorem 2). Here mathematical measures emphasize that we see statistically higher 4-weighted robustness to ±5% input noise in Tanaka cooling and LS methods ( Table 8): where error propagation occurs in Tanaka and LS methods, and there is no aid to the degrees of freedom since mathematically there is inherent failure. When the Lipschitz contraction condition L < 1 has been achieved.

### Practical implications for engineering

The implications of these developments to uncertainty-consciousness in material science and structural engineering are immense. In high stakes tasks, such as concrete strength prediction, our method will not only provide useful point estimates, but also provide estimate probabilities in the form of quantifiable uncertainty bounds, depending on the width of the TrFNs (e.g., 32.7–37.3 MPa, in one particular 35 MPa sample). This allows engineers to spread inaccuracy in the calculation of design, to assist in the decision-making that is directed by reliability. As an example, the 18.3% ambiguity elimination is a direct translation of smaller safety margins in the load-bearing calculation, which may result in a 12–15% decrease in the overdesign of the materials with the same level of safety (ACI 318–19).

### Limitations and methodological considerations

There are three constraints that should be mentioned. First, the contraction property depends on Lipschitz condition *K*·max∥ $\tilde{X}$ ∥ < 1 that might require data scaling in high-magnitude predictors (e.g. aggregate content more than 1,000 kg/m ^3^). Second, TrFNs are effective in the modeling of symmetric uncertainty but might need a modification to skewed distributions involving Gaussian or LR-type fuzzy numbers. Third, computational complexity is linear in predictors ( *O* ( *np*) but for *p* > 50, gradient updates by iteration ( Equation 7) can be accelerated with quasi-Newton methods.

Importantly, performance is determined by the right fuzzification parameters ( Table 5). An excessively narrow support (i.e., *δ*_2_ < 2%) suppresses uncertainty whilst allowing too broad supports to exaggerate ambiguity. We suggest finding *δ*_1_, *δ*_2_ delay A domain-specific standards (e.g. ASTM tolerances) or resampling on the boots. Further studies into automated fuzzification through uncertainty quantification algorithms such as Monte Carlo dropout should be undertaken in the future.

### Synthesis

This paper shows that fixed-point theory does not just formulate an elegant theory on paper but it also provides a real application of fixing the practicality of the fuzzy regression concept. Introducing estimation into a full metric space, and exploiting the Banach contractive properties, we complete the existence-uniqueness-stability trilemma, which has plagued the subject since Tanaka introduced it, and which had earlier impeded the development of the subject. The resulting framework balances mathematical rigor and engineering utility a pivotal measure to reliable quantification of uncertainty in data-driven design.

## Conclusion

This paper presents a mathematically rigorous framework for fuzzy regression based on fixed-point theory in the complete metric space of trapezoidal fuzzy numbers under the (d _∞_)-metric. Unlike classical fuzzy regression models, such as Tanaka’s minimum-fuzziness approach and least-squares-based methods, the proposed framework guarantees the existence, uniqueness, and convergence of the estimated solution through Banach’s Fixed-Point Theorem under verifiable Lipschitz conditions.

Our strict convergence criterion iterative algorithm was developed and effectively implemented in Python. Empirical validation on the UCI Concrete Compressive Strength Dataset demonstrated that one can obtain substantially better predictive level (the reduction in the MSE is 12.5%) and less uncertainty (the ambiguity of the parameters is dropped by 18.3%) than the ones available in benchmarking methods. It was also shown that the model was highly robust to noise perturbation and this would be suitable in the real-world engineering situation.

The paper provides a solid mathematical ground on fuzzy regression in case of uncertainty and justifies its use in data-driven fields. Further applications Future applications Future studies will include extensions to non-trapezoidal fuzzy numbers (e.g. Gaussian, LR-shaped), nonlinear model structures, fuzzification by machine-learning, and their application to economics and biomedical application.

## Data Availability

All data supporting the findings of this study are openly available in Zenodo under a CC-BY 4.0 license. The dataset includes fuzzified trapezoidal data, numerical values underlying all tables and figures, simulation outputs, and extended materials. The complete dataset and extended data package can be accessed at: https://doi.org/10.5281/zenodo.17772389 (Abdalrahem, M. (2025).) ^3^
