# Supplementary material for: Employing Fixed-Point Theory for Fuzzy Regression Analysis: Methodology and Empirical Application
Source: F1000Res. 2026 Jul 27;14:1435. Originally published 2025 Dec 23. [Version 2] doi: 10.12688/f1000research.173734.2 (PMC13560055; doi:10.12688/f1000research.173734.2)
Supplement: Peer review history

# Reviewer report: Reviewer response for version 2

**DOI:** 10.5256/f1000research.198592.r506325

**Published:** 2026-08-24

**Authors:** Heng Xia

**Affiliations:** 1 Beijing University of Technology (Ringgold ID: 12496), Beijing, Beijing, China

This manuscript proposes a fixed-point-theoretic framework for trapezoidal fuzzy linear regression, providing conditional guarantees of existence, uniqueness, and convergence of the estimated fuzzy coefficients. The manuscript has been substantially improved compared with the previous version. However, several minor issues should be further clarified to improve its rigor and presentation. The detailed comments are as follows:

1. The guarantees of existence, uniqueness, and convergence are established under the condition L<1, where L may depend on multiple factors. Thus, the theoretical guarantees should be understood as conditional rather than universal. Although this point is acknowledged in the limitations section, the authors are encouraged to clarify this point more explicitly in the statements of the main contributions and conclusions.
2. The selected core and support widths δ_1 and δ_2 is important for the fuzzy input/output construction, but their sensitivity has not been investigated. Please provide a sensitivity analysis or further justify the selected values, particularly by clarifying their relationship with the cited ASTM/ACI standards and the corresponding uncertainty percentages.
3. The current MSE evaluation reduces each predicted TrFN to the midpoint of its core, thereby ignoring its support and uncertainty information. Please provide a clearer justification for this scalarization, particularly considering that uncertainty representation is a key feature of fuzzy regression.

I hope these comments and suggestions will help improve the quality of the manuscript. Thanks.

Is the work clearly and accurately presented and does it cite the current literature?

Yes

If applicable, is the statistical analysis and its interpretation appropriate?

Partly

Are all the source data underlying the results available to ensure full reproducibility?

Yes

Is the study design appropriate and is the work technically sound?

Yes

Are the conclusions drawn adequately supported by the results?

Partly

Are sufficient details of methods and analysis provided to allow replication by others?

Yes

Reviewer Expertise:

data-driven modeling

I confirm that I have read this submission and believe that I have an appropriate level of expertise to confirm that it is of an acceptable scientific standard, however I have significant reservations, as outlined above.

# Reviewer report: Reviewer response for version 2

**DOI:** 10.5256/f1000research.198592.r505378

**Published:** 2026-08-18

**Authors:** Vahid Shafaie

**Affiliations:** 1 Széchenyi István University, Győr, Hungary

Thanks authors for their effort to address most comments.

Is the work clearly and accurately presented and does it cite the current literature?

Partly

If applicable, is the statistical analysis and its interpretation appropriate?

Partly

Are all the source data underlying the results available to ensure full reproducibility?

Partly

Is the study design appropriate and is the work technically sound?

Yes

Are the conclusions drawn adequately supported by the results?

Yes

Are sufficient details of methods and analysis provided to allow replication by others?

Partly

Reviewer Expertise:

Concrete, Numerical modeling, AI (Machine learning and Deep Learning), Fuzzy system,

I confirm that I have read this submission and believe that I have an appropriate level of expertise to confirm that it is of an acceptable scientific standard.

# Reviewer report: Reviewer response for version 1

**DOI:** 10.5256/f1000research.191573.r450666

**Published:** 2026-01-28

**Authors:** Areej Tawfeeq Hameed

**Affiliations:** 1 University of Baghdad, Baghdad, Baghdad Governorate, Iraq

Hello,

 ​I have reviewed the submitted manuscript and find that the paper is highly suitable for publication. The authors have presented several appropriate and mathematically sound results that contribute significantly to the current field of study. Furthermore, the practical implications of this research produce useful applications for society. I recommend its acceptance as it meets the necessary academic standards for this journal.

 ​Prof. Dr. Areej Tawfeeq Hameed"

Is the work clearly and accurately presented and does it cite the current literature?

Yes

If applicable, is the statistical analysis and its interpretation appropriate?

I cannot comment. A qualified statistician is required.

Are all the source data underlying the results available to ensure full reproducibility?

Partly

Is the study design appropriate and is the work technically sound?

Yes

Are the conclusions drawn adequately supported by the results?

Yes

Are sufficient details of methods and analysis provided to allow replication by others?

Yes

Reviewer Expertise:

math.

I confirm that I have read this submission and believe that I have an appropriate level of expertise to confirm that it is of an acceptable scientific standard.

# Reviewer report: Reviewer response for version 1

**DOI:** 10.5256/f1000research.191573.r448782

**Published:** 2026-01-28

**Authors:** Vahid Shafaie

**Affiliations:** 1 Széchenyi István University, Győr, Hungary

**Overall Evaluation**

 The manuscript proposes a mathematically grounded fuzzy linear regression framework that embeds trapezoidal fuzzy-number (TrFN) regression in the complete metric space induced by the *d*∞ metric and estimates fuzzy coefficients via a Banach fixed-point (contraction mapping) formulation. The central contribution is the introduction of a provably unique and convergent estimation scheme for fuzzy regression coefficients—an important advance over classical Tanaka-type linear programming and fuzzy least-squares variants that may suffer from ambiguity and instability—followed by an empirical demonstration on the UCI Concrete Compressive Strength dataset with reported improvements in prediction error, coefficient ambiguity, and robustness under ±5% noise. The work is timely and potentially impactful for uncertainty-aware engineering prediction (notably concrete strength), and the manuscript is generally well-structured with relevant theoretical and empirical components. However, several technical, interpretive, and presentation aspects are insufficiently clear or underdeveloped and should be addressed as outlined below.

**Specific comments:**

1. What is the *exact* definition of the contraction operator *T* and the objective *J(θ)* in fully explicit mathematical form (including the precise predicted output expression and how *d*∞ enters the loss)? Please provide the full equations as implemented, not only verbal descriptions.
2. The text states the method “avoids explicit gradient computation,” yet the algorithm and complexity table indicate gradient computation and an update step using ∂ *J*/∂ *A* *j* . Which is correct? If gradients are computed, specify how derivatives/subgradients are defined under *d*∞ (a non-smooth metric) and how this is reconciled with the fixed-point formulation.
3. The contraction condition is pivotal. How were predictors scaled/normalized to ensure *L<1* in practice, and is this condition guaranteed across datasets or only for the current scaling? Please report the exact preprocessing, the step-size/relaxation parameter (if any), and a formal statement of when the method may fail (e.g., *L≥1* ).
4. How is MSE computed for fuzzy outputs? The manuscript mentions evaluation at *α=0* and *α=1* . Do you compute MSE on (i) the support endpoints, (ii) the core endpoints, (iii) the *d*∞ distance, or (iv) another scalarization? Provide the precise formula(s) and justify why this is the appropriate accuracy metric for fuzzy predictions.
5. How exactly were Tanaka’s minimum-fuzziness and the LS-based fuzzy regression implemented (software/package, constraint formulation, solver settings, handling of infeasibility/divergence)?
6. The manuscript should briefly position its fixed-point TrFN fuzzy regression (with existence/uniqueness/convergence guarantees) against the mainstream *applied* fuzzy-logic practice in concrete materials research, where rule-based fuzzy inference systems are integrated with experimental bond-test programs. In particular, the recent studies “Slant Shear Tests and Fuzzy Logic Integration for Evaluating Shear Bond Strength in SCC and FRSCC Repair Applications (2025)” and “Integrating push-out test validation and fuzzy logic for bond strength study of fiber-reinforced self-compacting concrete (2024)” exemplify how fuzzy logic is used for SCC/FRSCC bond-strength prediction and test-method validation; the authors should add a short paragraph clarifying what their approach adds relative to such FIS-based studies (and what it does not), and whether the framework is readily extendable beyond compressive strength to bond-related targets.
7. Precisely define “ambiguity” (e.g., average coefficient trapezoid width? output interval width? aggregated across coefficients?) and give the computation formula. Clarify whether Table 7 width values refer to coefficients or predicted outputs, and whether widths are measured at *α=0* , *α=1* , or across all *α* .
8. Tables show fuzzification parameters for only some variables in one table while another includes additional variables. Provide a complete fuzzification table covering *all* predictors and the response, with sources/justifications for each *δ* *1* *δ* *2* . Explicitly state whether the response is fuzzified during training and how.
9. The manuscript contains systematic reference-number mismatches (e.g., key foundational citations appear misaligned with the bibliography). This must be fully corrected so every in-text citation points to the correct source; also ensure standards referenced in text (ACI documents, ASTM standards) are properly and consistently cited.

**Decision**

**Minor Revision**

 The manuscript’s core idea—casting fuzzy regression estimation as a contraction mapping in a complete TrFN *d*∞ -metric space with a unique fixed point—is novel and technically promising, and the reported empirical outcomes are consistent with the stated objectives. Nevertheless, several issues must be corrected before the work is suitable for indexing. Addressing the numbered items above should be sufficient to make the manuscript verifiable, technically unambiguous, and editorially sound.

 =====

 · **Is the work clearly and accurately presented and does it cite the current literature?**

**Partly** (presentation is generally structured, but citation-numbering integrity and some clarity issues must be corrected; literature coverage is mostly current but referencing is unreliable until fixed).

 · **Is the study design appropriate and is the work technically sound?**

**Yes** (the theoretical framework and empirical benchmarking design are appropriate; the core approach appears technically sound).

 · **Are sufficient details of methods and analysis provided to allow replication by others?**

**Partly** (key elements—explicit definition of *T* , exact loss/MSE computation for fuzzy outputs, preprocessing/scaling choices, baseline implementation details, and statistical testing protocol—need clearer specification).

 · **If applicable, is the statistical analysis and its interpretation appropriate?**

**Partly** (reporting p-values is positive, but the protocol generating them—repetitions/splits/tests—must be explicitly documented to validate interpretation).

 · **Are all the source data underlying the results available to ensure full reproducibility?**

**Partly** (a data package is indicated, but full reproducibility requires confirmation that code, preprocessing/fuzzification scripts, and baseline solver settings are available; otherwise it remains partial).

 · **Are the conclusions drawn adequately supported by the results?**

**Yes** (within the scope of the presented dataset and tests, the conclusions align with the reported results; claims should be carefully scoped to avoid overgeneralization).

Is the work clearly and accurately presented and does it cite the current literature?

Partly

If applicable, is the statistical analysis and its interpretation appropriate?

Partly

Are all the source data underlying the results available to ensure full reproducibility?

Partly

Is the study design appropriate and is the work technically sound?

Yes

Are the conclusions drawn adequately supported by the results?

Yes

Are sufficient details of methods and analysis provided to allow replication by others?

Partly

Reviewer Expertise:

Concrete, Numerical modeling, AI (Machine learning and Deep Learning), Fuzzy system,

I confirm that I have read this submission and believe that I have an appropriate level of expertise to confirm that it is of an acceptable scientific standard, however I have significant reservations, as outlined above.
